# Silk-Derived Antibacterial Hydrogels: Material Identity, Mechanistic Evidence, and Translation

**DOI:** 10.3390/gels12090809

**Published:** 2026-09-03

**Authors:** Hongmei Wang, Bingbing Xia, Yanlin Zhang, Xiaojuan Mi

**Affiliations:** 1 School of Basic Medicine, Shaanxi University of Chinese Medicine, Xianyang 712046, China; 18717296750@163.com; 2 The Second School of Clinical Medicine, Shaanxi University of Chinese Medicine, Xianyang 712046, China; xiabingbing1886@163.com

**Keywords:** silk fibroin, silk sericin, antibacterial hydrogel, biofilm, infected wound, drug delivery, photothermal therapy, nanozyme, environmental remediation

## Abstract

Silk fibroin (SF)- and silk sericin (SS)-based antibacterial hydrogels are increasingly engineered as local antimicrobial platforms, yet cross-study interpretation is limited by inconsistent material reporting and by conflation of bacterial inhibition with tissue repair. We performed a structured evidence-mapping and critical synthesis of a frozen 2020–July 2026 corpus of 94 references. The original 46-record core map was re-audited at the original-article level: 43 full-text-verified, non-retracted primary studies were retained for detailed evidence grading, 2 records available only at abstract/database level were retained descriptively but not graded, and 1 subsequently retracted study was excluded from quantitative synthesis. Among the 43 graded studies, metal-ion/nanozyme/catalytic systems were most common (12/43, 27.9%), followed by release-mediated (11/43, 25.6%), multimodal (9/43, 20.9%), contact-active/anti-adhesive (6/43, 14.0%), and light-responsive systems (5/43, 11.6%). Sixteen studies (37.2%) used deliberately infected animal models, whereas only 4 (9.3%) reached a biofilm or adherent-bacteria-level endpoint in the graded map. Biological claim ceilings (C0–C5) are assessed independently from translation gates spanning material identity, reproducibility, mechanism, host safety, sterilization/storage, resistance, long-term fate, and deployment. Across mechanisms, SF and SS most often function as structural, interfacial, or transport-regulating matrices; direct silk-dependent bactericidal causality remains uncommon. The central translational deficit is failure to quantitatively link silk molecular identity and network architecture to antimicrobial exposure, bacterial killing, host selectivity, and long-term material fate.

## 1. Introduction

Antibacterial hydrogels have developed from passive moisture-retaining dressings into multifunctional systems intended to control pathogens, modulate inflammation, and support tissue regeneration. Within this field, silk fibroin (SF) has been reviewed as a versatile antibacterial biomaterial and hydrogel platform, while silk sericin (SS) has increasingly been considered as a bioactive component for wound-oriented composites [1,2,3,4,5,6]. Broader analyses of silk chemistry and biomedical hydrogel design emphasize that silk proteins are unusually processable across films, fibers, particles, porous scaffolds, and three-dimensional networks [7,8].

The two principal silk proteins are functionally distinct and should not be treated as interchangeable matrix labels. SF is dominated by repetitive Gly-Ala/Ser-rich motifs whose hydrophobic packing enables β-sheet-rich crystalline domains; its molecular-weight distribution and β-sheet fraction strongly influence chain entanglement, gel stability, degradation, mesh size, and payload diffusion. SS is more hydrophilic and compositionally heterogeneous, with abundant polar and ionizable residues and processing-sensitive molecular-weight fractions that alter charge, water uptake, functional-group availability, metal/polyphenol interactions, and interfacial transport [5,7,9,10]. Degumming severity, sericin extraction, dissolution, dialysis, storage, residual sericin, and sterilization therefore change the material being tested rather than merely the preparation details [9,10,11]. Accordingly, any antibacterial comparison must distinguish an SF structural/transport effect from an SS hydrophilic/interfacial effect and from the direct activity of an incorporated antimicrobial component.

The antibacterial performance of a silk-derived hydrogel is therefore not determined solely by the presence of an antimicrobial additive. Network density, swelling, protein conformation, degradation, and active-group accessibility determine whether an antimicrobial agent is released rapidly, remains immobilized at the interface, becomes catalytically activated, or requires an external trigger. The same design variables also affect mammalian-cell exposure and tissue integration. Existing reviews have summarized antibacterial additives and wound-healing applications [1,2,3,6], but a systematic appraisal connecting material identity, network transport, antimicrobial evidence, and the maximum defensible biological claim remains limited.

The Special Issue context also raises a second boundary. Silk is a renewable and biodegradable feedstock, and silk-based materials have been explored in water treatment and sustainable remediation [11,12]. Nevertheless, direct evidence for silk-derived antibacterial hydrogels in environmental applications is far less developed than the biomedical literature. Treating tissue engineering and environmental disinfection as equally mature fields would therefore overstate the evidence.

**Figure 1:**
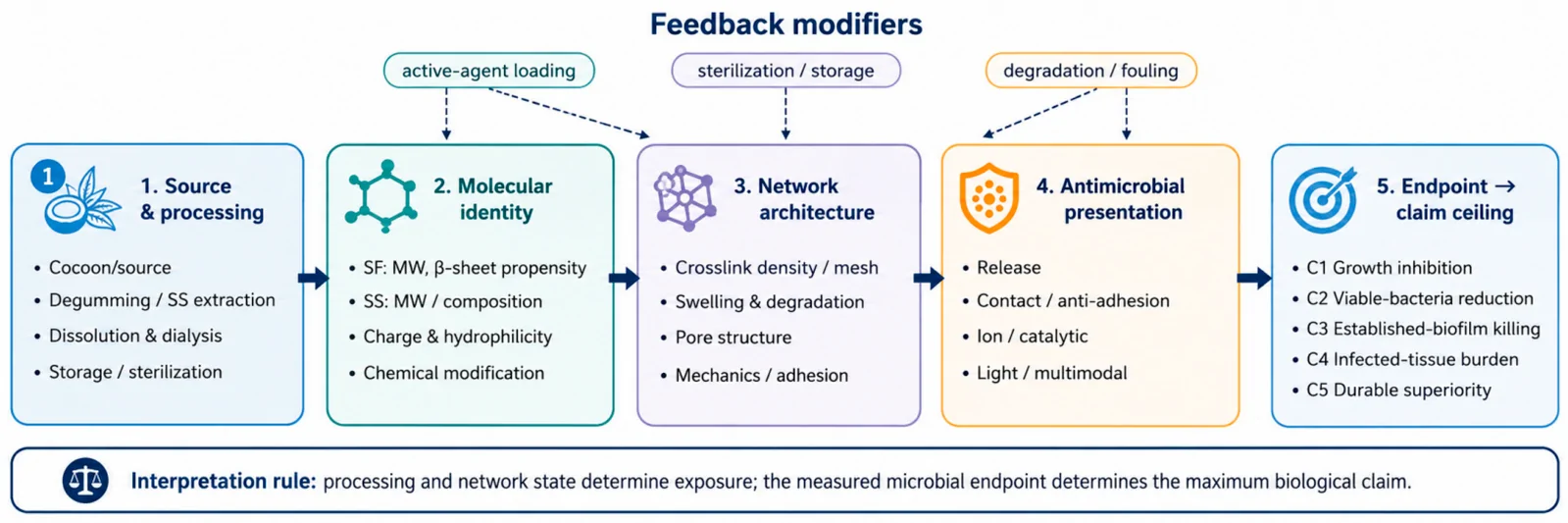
Revised source–structure–mechanism–outcome framework. Silk source and processing determine molecular identity; molecular identity and crosslinking determine network architecture; the network controls antimicrobial presentation; and the directly measured microbial endpoint sets the biological claim ceiling. Solid arrows indicate the forward source–identity–network–presentation–endpoint sequence, whereas dotted arrows indicate feedback modifiers showing how active-agent loading, sterilization/storage, and degradation/fouling can alter network state, exposure, efficacy, and toxicity.

For terminology consistency, ‘silk-derived antibacterial hydrogel(s)’ is used hereafter as the umbrella term for systems in which SF and/or SS is a load-bearing, crosslinking, or indispensable structural phase; protein-specific terms such as ‘SF hydrogel’ and ‘SS hydrogel’ are retained only when the composition or mechanism specifically concerns that protein. This review makes the source–structure–mechanism–outcome framework the central analytical model. The quantitative map first asks what silk material was actually produced, then how its molecular state and crosslinking architecture regulate antimicrobial presentation, which microbial endpoint was directly measured, and therefore what maximum biological claim is defensible. Biomedical and environmental translation are evaluated through separate gates rather than being placed on the same evidence ladder. The objective is not to catalog increasingly complex formulations, but to identify which silk-specific material variables measurably improve antimicrobial exposure or durability, which effects remain additive-driven, and where evidence fails before clinical or environmental deployment. The overall analytical framework is summarized in Figure 1.

## 2. Review Design, Search Verification, and Evidence Mapping

The revised article is defined as a structured evidence-mapping and critical synthesis rather than a prospectively registered systematic review or a meta-analysis of intervention efficacy. The original review was assembled retrospectively, and raw hit counts from the earliest discovery searches were not archived. To avoid retroactively inventing PRISMA identification counts, the revision reports a reproducible verification strategy for the frozen evidence set and an evidence-classification flow in the Supplementary Materials. In August 2026, PubMed/MEDLINE was the sole formal bibliographic database used for retrospective re-verification, supplemented by DOI-linked original-article sources and backward citation chaining from silk-specific reviews. A second database was not added after the evidence set had been frozen because doing so without re-running discovery, deduplication, screening, and eligibility from inception would create a hybrid workflow that could misleadingly imply prospective multi-database coverage; this single-database design is explicitly treated as a limitation. The verification query combined three concept blocks: (silk fibroin OR sericin OR silk protein OR SilkMA OR SilMA) AND (hydrogel OR cryogel OR injectable OR photocrosslink*) AND (antibacter* OR antimicrobial* OR biofilm OR infected wound OR wound infection OR disinfection). The exact fielded search string is supplied in Supplementary Methods.

The quantitative core map was intentionally restricted to January 2020 through July 2026 to capture contemporary silk-derived antibacterial hydrogel architectures, including photocrosslinkable, catalytic, nano-enabled, and multimodal systems. This cutoff is a scope decision rather than a claim that earlier work is unimportant; pre-2020 foundational studies can inform material chemistry and historical context but are not counted in the 43-study graded quantitative map. Primary studies were eligible when SF or SS was a load-bearing network component, a critical crosslinking component, or an indispensable structural phase of the final hydrogel, and at least one direct microbial endpoint was reported. Studies in which sericin served only as a nanoparticle template, or silk was absent from the final load-bearing network, were excluded from the core set. Supporting primary studies were retained for material identity, crosslinking, mechanics, degradation, transport, tissue repair, sterilization, or environmental context even when they lacked a direct microbial endpoint.

The frozen evidence base comprises 94 references: 46 primary records originally eligible for the antibacterial core, 34 supporting primary silk-derived hydrogel studies, and 14 silk-specific reviews or methodological sources. During the second-round audit, all 46 original core records were re-opened at the original-source level. One record was found to have been formally retracted and was excluded from all quantitative synthesis and evidence grading. Two records could be verified only at abstract/database level in the available audit trail; they remain visible for transparency but are not used for detailed claim-ceiling or silk-contribution grading. The detailed graded map therefore comprises 43 full-text-verified, non-retracted primary studies. For these 43 studies, we extracted mechanism class, silk identity and role, crosslinking, active antimicrobial component, microorganism, microbial assay, biofilm category, animal-infection design, host-safety information, release or activation information, and major translational gaps. Numerical values were retained only when directly reported and meaningfully interpretable; otherwise the Supplementary evidence map uses NR rather than harmonizing incompatible concentrations, geometries, units, or assay conditions. Quantitative synthesis is descriptive: 12/43 (27.9%) metal-ion/nanozyme/catalytic, 11/43 (25.6%) release-mediated, 9/43 (20.9%) multimodal, 6/43 (14.0%) contact-active/anti-adhesive, and 5/43 (11.6%) light-responsive.

Biological evidence and translation maturity are now scored on orthogonal axes. The biological claim ceiling is C0, material functionality without a direct microbial effect; C1, growth inhibition; C2, quantitative reduction in viable bacteria; C3, reduction in viability of a pre-established biofilm; C4, quantitative reduction in bacterial burden in deliberately infected tissue; and C5, durable or superior antimicrobial control versus an appropriate standard-of-care or exposure-matched comparator with longitudinal or recurrence evidence. Anti-adhesion, prevention of biofilm formation, biofilm disruption, and biofilm killing are annotated separately and do not automatically qualify as C3. Translation is assessed separately through eight gates: T1 material identity/batch reproducibility, T2 exposure and quantitative efficacy, T3 mechanism-specific causality, T4 host selectivity, T5 sterilization, T6 storage stability, T7 resistance/adaptation plus long-term fate or leaching, and T8 large-animal, clinical, or environmental deployment. This separation prevents an infected animal study from being misread as manufacturing or clinical maturity.

## 3. Silk Material Identity and Hydrogel Network Design

### 3.1. Silk Fibroin Source, Processing, and Structural State

Regenerated SF is usually produced by degumming cocoons to remove sericin, dissolving the remaining fibroin, dialyzing the solution, and inducing network formation through physical, chemical, enzymatic, or photoactivated processes. Reviews of silk structure and extraction emphasize that these steps alter chain length, crystalline propensity, solubility, and biological performance [7,9,11]. Primary hydrogel studies further show that ultrasonication, concentration, processing time, and one-step physical assembly affect gelation time, rheology, drug-release behavior, and mechanical strength [13,14,15].

Beta-sheet formation is central to many physically crosslinked SF hydrogels. Increased beta-sheet content generally stabilizes the network and reduces dissolution, but the resulting effects on swelling and payload transport depend on chain length, concentration, and composite formulation. A natural SF drug-delivery hydrogel demonstrated that preparation history can alter rheological and release properties [15], while robust one-step silk-protein networks and ultrasonication-induced injectable gels illustrate distinct routes to structural consolidation [13,14]. These studies support the treatment of processing history as a design variable rather than a reproducible background procedure.

Chemical modification expands the available network window but changes the protein identity. Methacrylated SF derivatives can be rapidly photocrosslinked and printed, yet substitution degree, initiator exposure, light dose, and residual reagents become additional critical quality attributes [16,17]. Irradiation can itself induce or modify SF gelation [18]. Accordingly, the designation “SF hydrogel” is insufficient for mechanistic comparison unless the preparation, molecular state, and crosslinking history are reported.

### 3.2. Sericin Extraction and Functional Heterogeneity

SS is not a single chemically uniform polymer. Cocoon source, pressure, temperature, extraction duration, pH, and purification change both molecular-weight distribution and the relative abundance/accessibility of polar, acidic, hydroxyl-, amino-, and aromatic side chains. These changes can alter net charge, hydration, metal coordination, polyphenol association, enzyme-mediated crosslinking, and diffusion of loaded compounds. Alternative silk sources and extraction methods substantially alter recovered sericin [10], while sericin reviews emphasize that biological and materials properties cannot be separated from processing history [5,6]. Thus, ‘SS-containing’ does not define a reproducible antibacterial material unless extraction and molecular state are reported.

Sericin has been incorporated into enzymatically crosslinked, physically crosslinked, ionic, and acid-induced networks. HRP-crosslinked in situ sericin hydrogels have been developed for wound repair [19,20], degraded sericin has been used to prepare sterile and injectable robust gels [21], and native sericin networks have been engineered for increased mechanical modulus [22]. Tannic-acid-containing HRP-crosslinked sericin gels and green physical crosslinking approaches further illustrate how functional and sustainability objectives can be integrated into network design [23,24].

**Figure 2:**
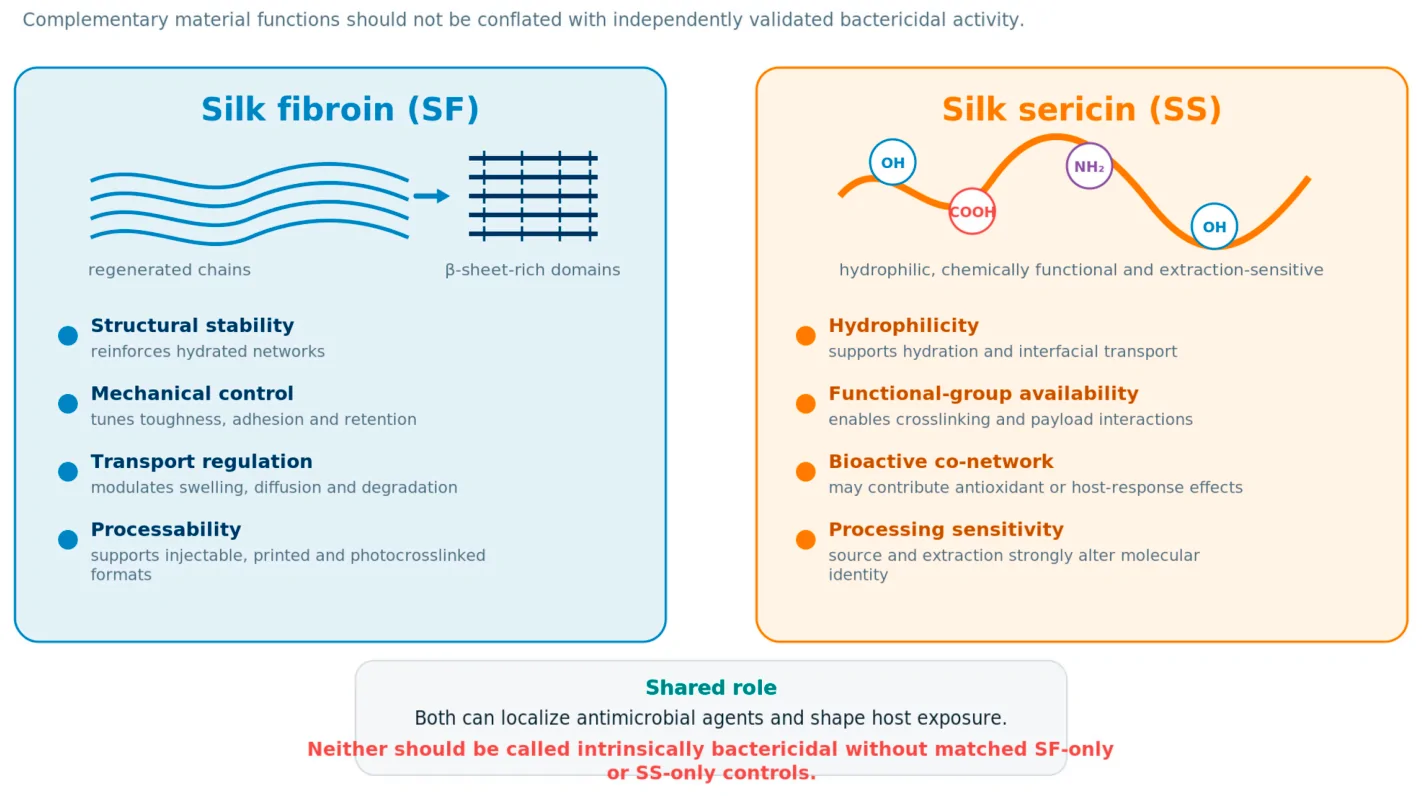
Functionally distinct roles of silk fibroin and silk sericin in antibacterial hydrogels. SF primarily contributes structural stability and transport control, whereas SS provides a hydrophilic and chemically functional network or co-network whose properties depend strongly on extraction history.

These studies support SS as a versatile hydrophilic network or co-network component, but they do not establish universal intrinsic bactericidal potency. By contrast with β-sheet-stabilized SF, SS more often contributes water uptake, interfacial accessibility, functional-group density, and compatibility with enzymatic or ionic crosslinking. Sericin-containing antibacterial hydrogels frequently rely on Ag, antibiotics, chitosan, polyphenols, or other active components. An intrinsic SS claim therefore requires an extraction-matched SS-only comparator and, ideally, a silk-free formulation that preserves the active additive exposure. The same logic applies to SF: structural or release-control contributions should not be relabeled as intrinsic killing. The complementary material roles of SF and SS are summarized in Figure 2.

### 3.3. Physicochemical Descriptors Linking Silk Identity to Antimicrobial Exposure

For SF, molecular weight and β-sheet content are coupled transport variables. More severe degumming or dissolution can shorten chains, reduce entanglement, and alter gelation; greater β-sheet organization generally increases physical stability and slows dissolution, but its effect on swelling and drug diffusion remains formulation-dependent [7,9,13,14,15,16]. Accordingly, a higher β-sheet fraction can plausibly reduce burst release by decreasing chain mobility and accessible aqueous pathways, yet the antibacterial consequence cannot be predicted without an actual release profile and microbial exposure measurement. Direct evidence that β-sheet content alone changes bacterial adhesion or mammalian-cell compatibility is sparse in the core antibacterial corpus.

Crosslink density should likewise be interpreted through mesh size rather than as a universally beneficial material property. Increasing crosslink density usually reduces swelling and diffusive mobility while improving mechanical retention; the same change can decrease antimicrobial exposure or prolong sublethal release. Macropore size and molecular mesh size must also be separated: micrometer-scale pores influence fluid transport, cell infiltration, and bacterial access, whereas nanometer-scale mesh dimensions govern diffusion of many dissolved payloads. Therefore, pore size should not be described as an antibacterial mechanism unless bacterial penetration, adhesion, or viable burden is measured directly.

For SS, hydrophilicity and ionizable functional groups can increase water uptake and chemical accessibility but do not inherently confer anti-adhesion or killing. The practical materials-science question is thus not whether SF or SS is ‘more antibacterial’, but whether a defined molecular state changes loading, release, interfacial presentation, or host selectivity at a fixed antimicrobial dose. This source-to-exposure relationship is the mechanistic bridge that remains least consistently quantified across studies.

### 3.4. Physical, Enzymatic, and Dynamic Crosslinking

Physical crosslinking avoids highly reactive covalent reagents and can preserve sensitive biological cargo, but it may produce slower gelation or greater formulation sensitivity. Ultrasonication, beta-sheet assembly, freeze–thaw cycling, ionic interactions, and physical polymer entanglement have all been used in silk-derived hydrogels [13,14,15,22,24]. Enzymatic crosslinking offers mild aqueous conditions and has been applied to SF-containing hyaluronic-acid networks, sericin hydrogels, and biofunctional polyphenol-containing systems [19,20,23,25].

Dynamic and interpenetrating networks are used when injection, self-healing, toughness, or wet adhesion is required. SF-polyacrylamide double networks, SF semi-interpenetrating networks, underwater-adhesive SF hydrogels, and native silk-fiber/sericin double-interpenetrating systems demonstrate how reversible interactions can be combined with permanent networks [26,27,28,29,30]. These architectures can improve retention at mobile or wet tissue surfaces, but greater network complexity can obscure which component controls antimicrobial transport.

### 3.5. Photocrosslinking, Microporosity, and Three-Dimensional Fabrication

Photocrosslinkable silk derivatives enable rapid in situ gelation, patterned fabrication, and integration with additive manufacturing. Riboflavin-mediated photocrosslinking, methacrylated SF, GelMA/SilMA composites, and dual-photopolymerized interpenetrating networks have been reported [16,31,32,33]. Digital light processing and bioprinting studies show that silk-containing networks can be fabricated with spatial control for cartilage, skin, and cell-delivery applications [34,35,36].

Microporous methacrylated SF networks and nanofiber-reinforced gelatin composites illustrate complementary strategies for increasing transport and mechanical stability [17,37]. Three-dimensional printed nerve-guidance conduits and conductive double networks further demonstrate the broad tissue-engineering range of silk-derived hydrogels [38,39]. These studies are not direct antibacterial evidence, but they define manufacturing approaches that could alter bacterial access, nutrient diffusion, immune-cell infiltration, and antimicrobial release in future systems.

### 3.6. Adhesion, Mechanical Reinforcement, and Composite Function

Wet adhesion and mechanical durability are important for wound dressings, sealants, implant interfaces, and dynamic tissues. Sweat-resistant SF-polyacrylamide networks, photocurable SF sealants, gastric adhesive semi-IPNs, spinal cord adhesive gels, and underwater adhesive SF networks demonstrate that adhesion can be engineered through multiple chemistries [28,29,30,40,41]. Chitosan/SF thermogels, conductive SF/GelMA conduits, and nanofiber reinforcement provide additional composite strategies [37,39,42].

Composite bioactivity does not necessarily imply antimicrobial activity. SF-Aloe vera hydrogels, sericin/PVA/carboxymethyl-cellulose films, resveratrol-loaded silk/sericin double networks, bFGF-delivery sericin gels, and platelet-rich-fibrin/sericin systems were developed primarily for material or regenerative functions [27,43,44,45,46]. They are relevant to network design and host repair, but they should not be cited as evidence of bacterial killing without direct microbial assays.

### 3.7. Minimum Material Reporting Requirements

A reproducible silk-derived antibacterial hydrogel should report silk species and source, degumming or sericin-extraction conditions, dissolution and dialysis, concentration, molecular-weight information where available, residual sericin, chemical modification, crosslinker and initiator concentrations, gelation time, secondary structure, swelling, degradation, pore structure, mechanical properties, sterilization, endotoxin control, and storage conditions. The minimum reporting framework used in this review is summarized in Table 1.

## 4. Mechanism-Based Antibacterial Strategies

Across the 43 full-text-verified, non-retracted primary studies, the dominant mechanisms were metal-ion/nanozyme/catalytic (12, 27.9%), release-mediated (11, 25.6%), multimodal/composite (9, 20.9%), contact-active/anti-adhesive (6, 14.0%), and light-responsive (5, 11.6%). Across categories, SF or SS was rarely the only bactericidal entity; silk most often formed or reinforced the hydrated network that localized antibiotics, metal species, cationic polymers, catalytic particles, or photoresponsive agents [47,48,49,50]. We therefore separate ‘silk contribution’ from ‘antibacterial determinant’: S0 denotes additive-dominated activity with silk serving primarily as matrix; S1 denotes a measured silk-assisted structural, release, or interfacial contribution; S2 denotes a silk-specific antimicrobial contribution supported by matched component controls; and S3 requires causal intrinsic silk-dependent killing. The present evidence is concentrated in S0–S1 rather than S3.

**Figure 3:**
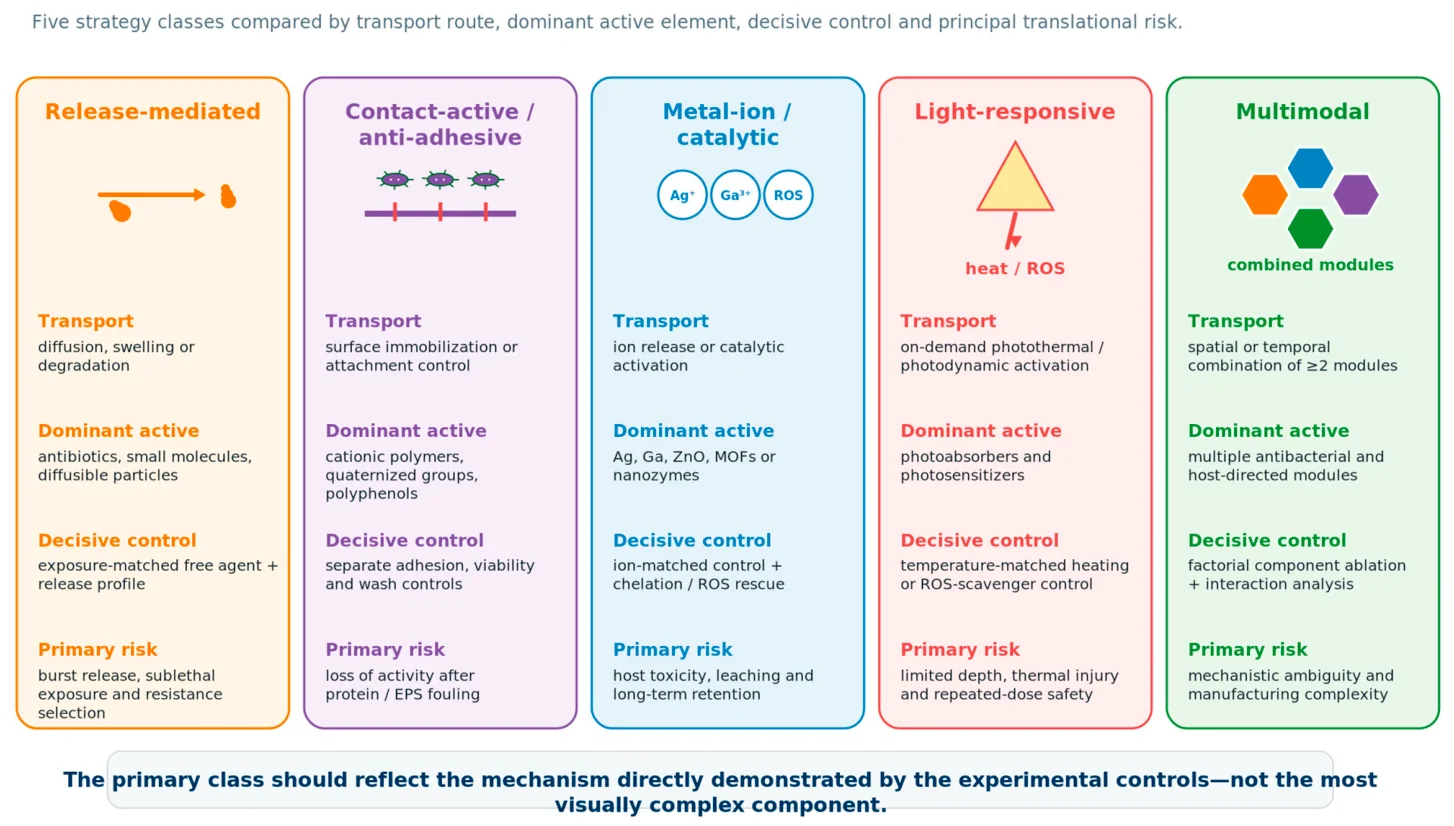
Antibacterial mechanism and transport matrix. Release-mediated, contact-active, metal/catalytic, light-responsive, and multimodal systems are compared according to transport route, bacterial target, required mechanistic control, and major translational risk.

The five mechanism classes are analytical rather than mutually exclusive. Classification is assigned by the mechanism directly tested, not by the most visually prominent component. Terminology is also endpoint-limited: inhibition zones or OD changes support C1; direct CFU/time-kill supports C2; a pre-established viable-biofilm assay can support C3; and only a deliberately infected animal model with quantitative tissue bacterial burden can support C4. Wound closure, histology, inflammation, or a surface swab cannot substitute for infected-tissue burden. The same principle applies to ‘eradication’ and ‘synergy’, which are reserved for below-detection/no-regrowth evidence and interaction-aware factorial comparisons, respectively. Figure 3 compares the five mechanism classes according to transport route, dominant active element, decisive control, and principal translational risk.

### 4.1. Release-Mediated Antibacterial Systems

Release-mediated systems use the silk-derived network as a local reservoir for antibiotics, natural antibacterial compounds, metal-containing particles, or other diffusible agents. Representative systems include rhein-loaded SF, Ag-containing photocrosslinked SF, azithromycin-containing SS/PVA or bacterial-cellulose/PVA composites, vancomycin-bearing injectable SF matrices, mupirocin-loaded SF, and ciprofloxacin-loaded acid-induced sericin hydrogels [48,51,52,53,54,55,56,57,58]. Their performance is governed not only by nominal loading but also by crosslink density, β-sheet formation, polymer–drug interactions, network degradation, and water uptake. Primary silk-derived hydrogel studies demonstrate that processing, methacrylation, physical gelation, and network architecture alter rheology, swelling, degradation, and release behavior [13,14,15,16,21]; however, the direction and magnitude of these effects remain formulation-specific rather than universally predictable.

Several formulations illustrate distinct transport regimes. Rhein, azithromycin, vancomycin, mupirocin, and ciprofloxacin are mainly presented as locally released antimicrobial agents [52,53,54,55,56,58]. By contrast, the Sil-MA/Ag/metformin platform was designed to separate an early antimicrobial phase from a later host-directed phase, with Ag-containing components providing initial bacterial inhibition and metformin-containing microspheres supporting subsequent immunomodulation [48]. These data support the concept that silk-derived antibacterial hydrogels can function as temporal delivery regulators, although few studies directly compared staged release with an exposure-matched single-phase regimen.

The strongest evidence in this category comes from systems that progressed beyond inhibition zones to adherent bacteria or biofilm-associated endpoints and infected animal models. Azithromycin-containing sericin composites and vancomycin-bearing SF matrices reached biofilm-associated or infection-related endpoints, while a vancomycin/quercetin-loaded SF scaffold was evaluated in chronic MRSA osteomyelitis [52,53,56]. The one-step sericin/ciprofloxacin hydrogel was also tested in an infected wound model [54]. Nevertheless, evidence quality remains heterogeneous: some studies reported infection-associated benefit without standardized tissue CFU, pathogen-specific longitudinal quantification, or a clearly described limit of detection [53,54,55,56], whereas others used non-inoculated burn or wound models and therefore primarily evaluated repair rather than treatment of an established infection [59].

In most antibiotic-containing systems, the released drug is the most plausible direct antibacterial determinant [52,53,54,56,57,58]. The contribution of the SF/SS network to bacterial killing cannot be separated from drug exposure unless the hydrogel is compared with an exposure-matched free-drug control and with a network lacking the active agent. Such controls were not consistently identifiable in the frozen evidence matrix [52,53,54,56]. Resistance selection was also not directly evaluated in the principal antibiotic-release studies, despite prolonged-release designs [52,53,54,56,58].

Manufacturing variables are especially relevant for release systems. Changes in SF molecular weight, β-sheet content, methacrylation, irradiation, or sericin degradation can modify gelation and transport behavior [13,14,15,16,18,21]. These observations identify a plausible source of batch-dependent exposure even when nominal drug loading is unchanged. The frozen set contains an SF hydrogel developed for environmental metal remediation [60], but it contains no primary study that directly measures antibiotic-selection pressure or antimicrobial leaching from an SF/SS antibacterial hydrogel in an environmental microbial community. Environmental resistance claims should therefore remain explicitly prospective.

Release-mediated systems are among the most controllable designs when local exposure is measured, because release profiles can be linked directly to CFU/time-kill and compared with an exposure-matched free agent. Their principal weakness is not lack of activity but incomplete pharmacokinetic–pharmacodynamic linkage and resistance testing; sustained sub-MIC exposure remains a specific risk for antibiotic-loaded systems.

### 4.2. Contact-Active and Anti-Adhesive Systems

Contact-active hydrogels are intended to damage bacteria at the material interface, whereas anti-adhesive systems aim to reduce attachment and subsequent biofilm development. SF/chitosan, SF/tannic-acid/quaternized-chitosan, particulate SF/Pluronic, antibiotic-free sericin, and SF/polyphenol systems represent this design space [61,62,63,64,65]. These endpoints must be distinguished experimentally because a reduction in recovered surface bacteria may reflect killing, weaker adhesion, detachment during washing, or reduced retention rather than a single bactericidal mechanism.

Direct interfacial activity is generally introduced through cationic polymers, quaternized groups, polyphenols, or other non-antibiotic active components [61,62,63,64,65,66,67]. SF commonly serves as a structural co-network that provides cohesion, wet stability, or mechanical reinforcement, whereas chitosan-derived cationic groups and polyphenols are the more plausible immediate antibacterial determinants [61,62,65]. In sericin-based antibiotic-free systems, antimicrobial activity was reported, but the contribution of sericin itself was not separated from that of the added active component [64,67].

Primary silk-material studies show that double-network formation, semi-interpenetrating networks, underwater adhesion, sericin crosslinking, and green physical crosslinking substantially alter swelling, mechanics, and network stability [22,23,24,28,29,30]. It is therefore reasonable to expect interfacial accessibility of cationic or phenolic groups to depend on network architecture, but this specific structure–antibacterial-interface relationship has rarely been measured directly. The particulate SF–Pluronic system is notable because it assessed adherent MRSA and reached a biofilm-associated evidence level, although the individual contributions of curcumin, Pluronic, and particulate architecture were not resolved [63].

Representative contact-active systems reported short-term mammalian-cell compatibility or hemocompatibility [61,62,63,64,65], but these assays do not establish long-term interfacial selectivity, irritation after repeated contact, or activity after adsorption of proteins and extracellular polymeric substances. No frozen primary study directly tested long-term antifouling performance of a silk-derived antibacterial hydrogel in an environmental matrix. The adjacent environmental SF hydrogel literature addresses contaminant capture rather than durable antimicrobial surface activity [60].

Contact-active and anti-adhesive designs are attractive when a non-leaching interface is desired, but adhesion reduction, surface killing, and mature-biofilm killing are distinct endpoints. Their main unresolved risks are protein/EPS fouling, loss of activity after repeated contact, and host-membrane toxicity for strongly cationic interfaces.

### 4.3. Metal-Ion, Nanozyme, and Catalytic Systems

Metal-containing and catalytic hydrogels constitute the largest mechanistic group in the frozen evidence base. Ag species, Ga^3+^, ZnO, metal–organic frameworks (MOFs), selenium nanoparticles, Mg(OH)_2_ nanorods, and catalytic nanozymes have been incorporated into silk-derived networks to produce ion-mediated growth inhibition, metabolic interference, ROS-associated killing, or catalytic conversion of endogenous substrates [47,49,50,59,68,69,70,71,72,73,74,75]. In these systems, the hydrogel can control both the transport of active species and the local catalytic microenvironment.

The structural role of silk differs markedly among formulations. Ag may be entrapped as nanoparticles, generated within a sericin-containing network, or used directly as a sericin crosslinker [47,49,50]. Ga^3+^ can participate in SF gelation while acting as an Fe3+ mimic that perturbs bacterial iron utilization [68,72]. MOFs can bridge or reinforce SF networks while carrying metal or catalytic functions [69,73,76], and nanozyme systems can generate or supply ROS-producing substrates within an SF matrix [71].

The principal mechanistic problem is distinguishing ion release, direct particle contact, catalytic ROS generation, and secondary network effects. Ag-containing hydrogels frequently combine more than one of these processes [47,49,50,59,75,77,78]. In the Ga-containing studies, growth inhibition was consistent with iron-metabolism interference, but iron-rescue experiments or direct bacterial iron-response measurements were not identified [68,72]. Likewise, catalytic activity and ROS-associated antibacterial effects were reported for MOF, ZnO, and nanozyme systems, but radical-scavenger rescue or matched catalytic-inhibition controls were not consistently available [69,70,71,73].

Evidence extends from simple in vitro inhibition to infection-associated animal models. Ga-containing SF systems, MOF-bridged networks, and catalytic nanozyme hydrogels reached higher evidence levels than many simple metal–particle composites [68,69,71,72]. However, several animal studies characterized wound closure, angiogenesis, inflammation, or collagen more extensively than quantitative tissue bacterial burden [49,50,59,68,69,72,73,75]. Thus, improved repair cannot be assigned solely to metal-mediated bacterial clearance.

Metal-based systems also have a narrow translational margin because antibacterial potency and host toxicity are influenced by the same variables: ion release, catalytic activity, ROS generation, and tissue retention. Representative studies generally reported short-term cytocompatibility or hemocompatibility, but not cumulative release, long-term organ retention, resistance adaptation, or ecotoxicity [49,50,59,70,71,73,74,75]. These untested risks are particularly relevant to environmental deployment, for which the frozen primary corpus contains no direct long-term leaching or microbial-community study of a silk-derived antibacterial hydrogel.

Metal-ion and catalytic systems show broad quantitative activity but have the narrowest margin between antibacterial exposure and host or environmental toxicity. Ion release, ROS dependence, and long-term retention therefore need mechanism-specific rescue and fate measurements before strong causal or translational claims are made.

### 4.4. Light-Responsive Systems

Light-responsive hydrogels use an external trigger to activate photothermal, photodynamic, or combined phototherapeutic effects. SF matrices have been used to disperse and retain Ag@reduced graphene oxide, liquid-metal phases, Au@MOFs, W18O49x@Au nanoparticles, and polydopamine-modified Ag/curcumin composites [76,79,80,81]. These systems provide temporal control because substantial antibacterial activity is intended to occur primarily during irradiation rather than continuously during storage or wear.

The silk network contributes by stabilizing photoactive phases, maintaining local hydration, and constraining nanoparticle mobility. SF coating was specifically used to stabilize a liquid-metal photothermal phase [79], while composite SF networks retained Ag@rGO, Au@MOF, W18O49x@Au, or PDA-modified particles [76,80,81]. Although these studies support effective incorporation and photoactivation, they do not provide a direct head-to-head comparison of how water content, oxygen diffusion, crosslink density, or nanoparticle distribution alters photothermal or photodynamic efficiency.

Light-responsive systems frequently reached infected-wound endpoints, including Ag@rGO, liquid-metal, Au@MOF, W18O49x@Au, and PDA-modified Ag formulations [76,79,80,81]. Nevertheless, the reported bacterial endpoints were not uniformly based on quantitative tissue CFU, and the depth and timing of sampling were often incompletely described. A designation of “infected-wound efficacy” should therefore not be interpreted automatically as demonstrated bacterial eradication.

Mechanistic attribution requires more than a light-versus-no-light comparison. Temperature-matched non-photothermal controls are needed to distinguish material-specific effects from nonspecific heating, while photodynamic claims require ROS measurements and preferably scavenger reversal. In Ag-containing photoresponsive systems, Ag release should also be measured independently because antibacterial activity may persist without irradiation [76,80,81]. The frozen evidence matrix did not identify a complete set of temperature-matched, ion-release-matched, and ROS-rescue controls in these representative systems.

The reviewed primary studies were evaluated mainly in superficial wound models and did not systematically test treatment depth, tissue pigmentation, necrotic burden, repeated irradiation, or long-term nanoparticle persistence [76,79,80,81]. No frozen primary study directly evaluated a photoresponsive silk-derived antibacterial hydrogel in turbid water or through repeated environmental disinfection cycles. Claims concerning deep-tissue penetration or environmental energy efficiency should therefore be presented as translational questions rather than established advantages.

Light-responsive systems provide unusually strong temporal control, but only if optical dose, temperature, oxygen/ROS, and particle release are measured independently. Their strongest evidence is concentrated in superficial infected-wound models; depth, pigmentation, repeated irradiation, and nanomaterial persistence remain practical constraints.

### 4.5. Multimodal and Composite Systems

Multimodal hydrogels combine antibacterial activity with functions directed at the changing wound microenvironment. Representative designs integrate staged Ag release with metformin-mediated immunomodulation, ε-polylysine-mediated membrane disruption with MnO_2_-dependent redox control, nitric oxide generation, oxygen production with a spatially separated Ag barrier, or antimicrobial treatment with visual monitoring [48,82,83]. These formulations are intended to address bacterial burden, hypoxia, oxidative stress, inflammation, hemostasis, and impaired repair within a single material platform.

SF and SS networks are well suited to spatial and temporal compartmentalization. Microspheres, nanoparticles, dual-region microneedles, and composite networks have been used to separate early antimicrobial functions from later oxygenation, immune modulation, or regenerative support [48,83]. The strongest direct example of spatial separation is the SilkMA microneedle system, in which oxygen-generating components and an Ag-containing antibacterial barrier were placed in different regions of the device.

The value of multimodal design depends on demonstrating that each module operates in the intended window and that the complete system outperforms its single-function components. The frozen evidence matrix did not identify complete factorial component-ablation designs for most representative systems [48,82,83]. Consequently, “synergy” was often inferred from the performance of the complete formulation rather than established by formal interaction testing. Host-repair endpoints such as macrophage phenotype, vascularization, collagen deposition, or wound closure were also generally characterized more extensively than quantitative bacterial burden [48,82,83].

Antibacterial and pro-regenerative effects should therefore be treated as independent evidence streams. A formulation may improve closure despite modest bacterial inhibition, while a strongly bactericidal formulation may impair repair if ROS, heat, ions, or cationic components damage host tissue. In visualizable sericin systems, the frozen evidence supports antimicrobial and wound-healing functions, but it does not yet establish that the visual signal quantitatively tracks bacterial burden rather than pH, exudate, inflammation, or other wound variables [83].

Multimodal systems also create substantial manufacturing complexity. Methacrylation, nanoparticle incorporation, irradiation, degraded-sericin processing, and multi-network assembly are all known to alter silk-derived hydrogel behavior [16,17,18,21,33]; combining several such modules increases the number of critical formulation and sterilization variables. However, the frozen primary studies did not directly compare manufacturing reproducibility or regulatory burden between simple and multimodal silk-derived antibacterial hydrogels. Environmental regeneration, component leaching, and end-of-life disposal were likewise not directly evaluated.

Multimodal systems offer the broadest functional coverage but the weakest attribution unless factorial component-ablation and exposure matching are performed. Their key translational liability is complexity: each additional module increases formulation, sterilization, reproducibility, and regulatory failure modes, so ‘more functions’ should not be treated as evidence of mechanistic synergy.

### 4.6. Cross-Mechanism Comparative Synthesis

The cross-mechanism comparison changes the practical ranking. Release systems and light-responsive systems are the most controllable in principle because exposure can be measured or externally switched, respectively. Metal/catalytic and multimodal systems have the broadest preclinical activity but also the greatest host-selectivity or attribution burden. Conventional antibiotic release carries the clearest resistance-selection risk, whereas non-antibiotic systems should not be called resistance-proof because adaptation to metals, oxidative stress, cationic surfaces, and repeated sublethal phototherapy remains biologically plausible and is rarely tested. For chronic wounds, the decisive discriminator is not mechanism label but performance against established biofilm plus quantitative infected-tissue burden. For environmental reuse, immobilized contact-active/anti-adhesive interfaces are conceptually more compatible than continuously leaching antibiotic or persistent-metal systems, provided activity survives organic fouling and regeneration.

No mechanism can currently be declared globally superior because matched head-to-head studies are absent. The most defensible field-level conclusion is that silk molecular identity and network architecture are upstream exposure variables, whereas the antimicrobial additive or trigger usually supplies the immediate killing force. Table 2 therefore replaces the previous study-by-study main-text list with a quantitative mechanism comparison; the audit of the original 46-record map and the 43-study graded evidence map, including study-level endpoint and claim-ceiling coding, is moved to Supplementary Table S1.

### 4.7. Biofilm Evidence: Five Non-Equivalent Claims

Biofilm terminology is standardized as follows: anti-adhesion means fewer initially attached cells; biofilm-formation inhibition means reduced biomass or viability when the material is present from inoculation; disruption means structural or biomass loss in an already established biofilm; biofilm killing requires a reduction in viable organisms within a pre-established biofilm; and eradication requires the strongest endpoint, ideally below-detection viability together with absent regrowth after material removal. These categories are coded separately in Supplementary Table S1. In particular, adhesion data and crystal-violet biomass alone do not justify ‘biofilm eradication’.

## 5. Biomedical Applications and Evidence Boundaries

### 5.1. Infected Acute and Chronic Wounds

Infected wounds are the dominant application of silk-derived antibacterial hydrogels. The evidence spans antibiotic-releasing gels, metal-ion systems, catalytic nanozymes, photothermal platforms, and multimodal matrices [53,54,55,72,76,80,83]. The shared rationale is to localize antimicrobial activity while maintaining a hydrated environment and supporting re-epithelialization, vascularization, or collagen remodeling.

A total of 16 of the 43 full-text-verified graded studies (37.2%) used deliberately infected animal models in the evidence map, but animal infection and C4 are not synonymous. Several studies emphasized wound closure, histology, inflammation, or macroscopic infection scores without quantitative tissue CFU. The revision therefore lists the animal model separately from the biological claim ceiling and assigns C4 only when deliberately infected tissue is sampled quantitatively. This change specifically prevents wound-healing improvement from being counted as bacterial clearance.

**Figure 4:**
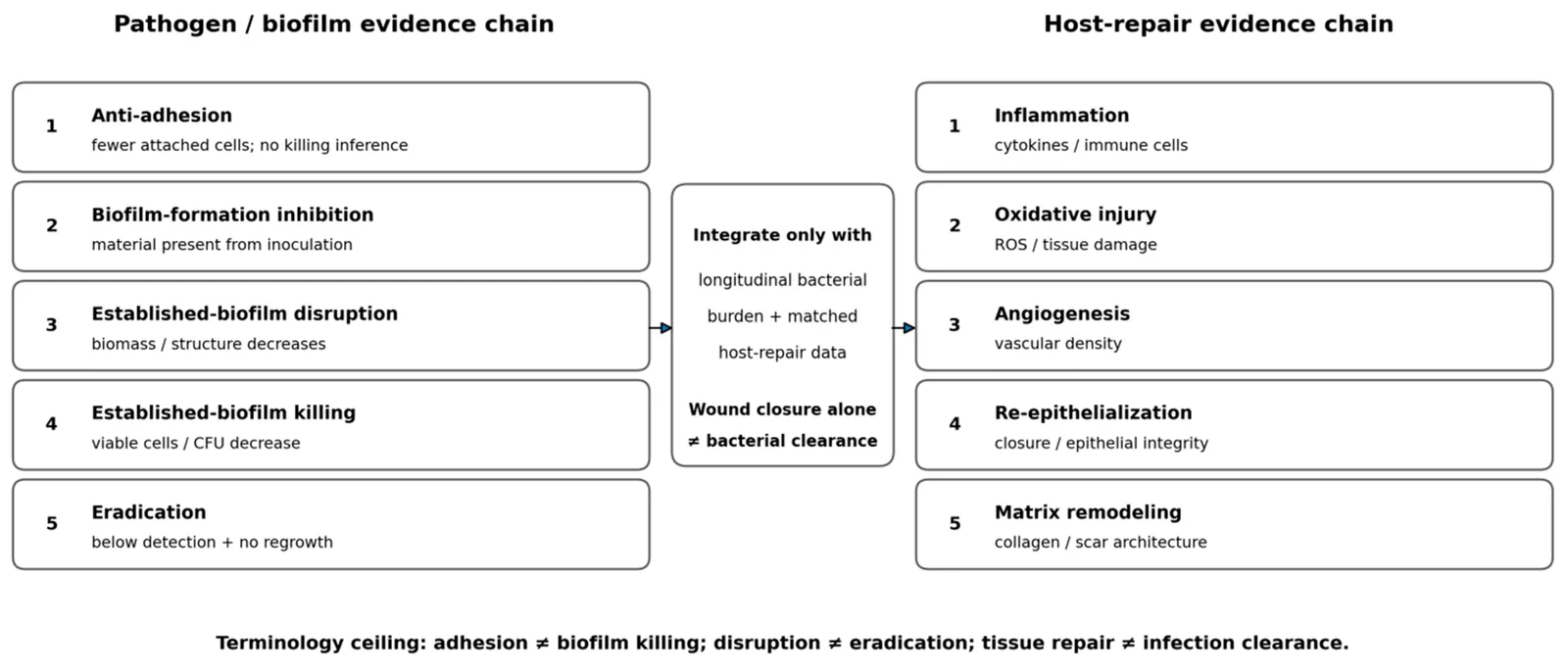
Biofilm terminology and separation of pathogen-control from host-repair evidence. Anti-adhesion, prevention of biofilm formation, disruption, viable killing, and eradication are non-equivalent claims. Inflammation, oxidative injury, angiogenesis, re-epithelialization, and matrix remodeling form a parallel host-response chain. The two chains should be integrated only when longitudinal bacterial-burden data are available.

This imbalance creates a recurrent causal risk. Faster closure can follow direct bacterial reduction, reduced inflammation, altered oxidative stress, improved oxygenation, or independent trophic effects of the hydrogel. A study that reports in vitro antibacterial activity and in vivo wound repair does not automatically demonstrate that bacterial clearance mediated the repair outcome. The two evidence streams should be presented separately unless longitudinal bacterial burden is measured. Figure 4 separates the pathogen-clearance and host-repair evidence chains.

### 5.2. Diabetic Wounds and Host-Microenvironment Modulation

Diabetic wounds combine microbial exposure with hypoxia, oxidative stress, vascular impairment, and dysregulated inflammation. Silk-derived systems have therefore been designed to provide sequential antimicrobial and immunomodulatory delivery, epsilon-polylysine-mediated killing with MnO_2_ redox regulation, oxygen-producing microneedles, Ag/polyphenol activity, and visual monitoring [48,64,83,84].

Supporting sericin studies show that SS networks can influence oxidative stress, hydration, growth-factor presentation, and repair in diabetic-wound settings even without a validated antibacterial endpoint [19,20,46,85]. This distinction is important: antioxidant or pro-regenerative activity can strengthen the therapeutic value of an antibacterial dressing, but it cannot substitute for bacterial measurement. Conversely, a strong antibacterial component may not correct the metabolic and vascular barriers that delay diabetic healing.

The highest defensible application claim is that selected silk-derived antibacterial hydrogels can combine an antibacterial function with host-microenvironment modulation in preclinical diabetic-wound models. Current evidence does not establish an optimal order or duration for bacterial killing, redox modulation, immune regulation, and angiogenic support.

### 5.3. Bone, Oral, and Implant-Associated Applications

Silk-derived antibacterial hydrogels have also been investigated for localized musculoskeletal or bone-related applications. Vancomycin/quercetin-loaded SF scaffolds were tested in chronic MRSA osteomyelitis, thermosensitive SF/calcium-phosphate/methylcellulose systems were developed for local vancomycin delivery, and Ag-containing SilMA nanocomposites combined antibacterial and osteogenic functions [56,57,59].

The broader tissue-engineering literature demonstrates the suitability of silk-containing thermogels, printed matrices, porous cell-delivery networks, nerve conduits, and conductive double networks [33,35,36,38,39,42,86]. These platforms provide manufacturing and biological precedents, but results from aseptic regeneration models should not be extrapolated to treatment of implant biofilms or infected defects. A bone-regeneration study with postoperative antibiotics, for example, cannot independently establish that an embedded antibacterial component cleared an established infection.

Translation in this area requires models that combine a clinically relevant implant or defect, a defined pathogen and inoculum, mature biofilm formation, quantitative bone and soft-tissue bacterial burden, mechanical integration, and systemic safety. The current literature usually addresses only part of this chain.

### 5.4. Drug Delivery and Regenerative Support Beyond Infection

Silk-derived hydrogels are established local delivery matrices for proteins, small molecules, and cells. Sericin/heparin systems have delivered bFGF, SF/chitosan thermogels have carried rhBMP-2, and resveratrol, platelet-rich fibrin, Aloe vera, and other regenerative cargoes have been incorporated into silk-derived networks [27,42,43,45,46]. These studies support silk as a delivery platform and inform release design for antibacterial systems.

However, regenerative cargo can complicate antibacterial interpretation. A formulation that contains an antibiotic and a trophic or anti-inflammatory agent may improve tissue outcomes through parallel rather than synergistic pathways. Factorial controls are required to determine whether the regenerative module improves bacterial clearance, merely mitigates host injury, or acts independently.

## 6. Environmental and Sustainable Antimicrobial Interfaces

Silk is renewable and potentially biodegradable, but renewable feedstock should not be equated with demonstrated sustainability of the finished antibacterial hydrogel. The antibacterial core literature is overwhelmingly biomedical; the environmental silk literature cited here mainly supports adsorption, separation, contaminant capture, or greener processing rather than direct pathogen inactivation [11,12,60]. We did not identify life-cycle assessment or techno-economic analysis that validates the overall sustainability of the primary silk-derived antibacterial hydrogel systems in the quantitative map.

Accordingly, environmental antibacterial use is treated as an emerging hypothesis rather than a co-equal mature application. The frozen primary corpus does not establish water-disinfection log reduction in complex matrices, durable membrane antibiofouling, repeated regeneration, community-level resistance effects, or long-term ecotoxicity for silk-derived antibacterial hydrogels. Claims are therefore restricted to demonstrated material processability and prospective translation opportunities.

Sustainability must be separated into at least five stages: raw-material sourcing and degumming/extraction; hydrogel synthesis and energy/solvent input; use-phase antimicrobial exposure; regeneration or reuse; and end-of-life fate. Green degumming or physical crosslinking can improve one stage without proving low life-cycle burden for a composite containing antibiotics, metals, MOFs, or photoactive nanoparticles. For environmental deployment, active-agent leaching, regeneration cycles, retained performance in organic matter/salinity, degradation products, and aquatic ecotoxicity are therefore mandatory rather than optional endpoints.

**Figure 5:**
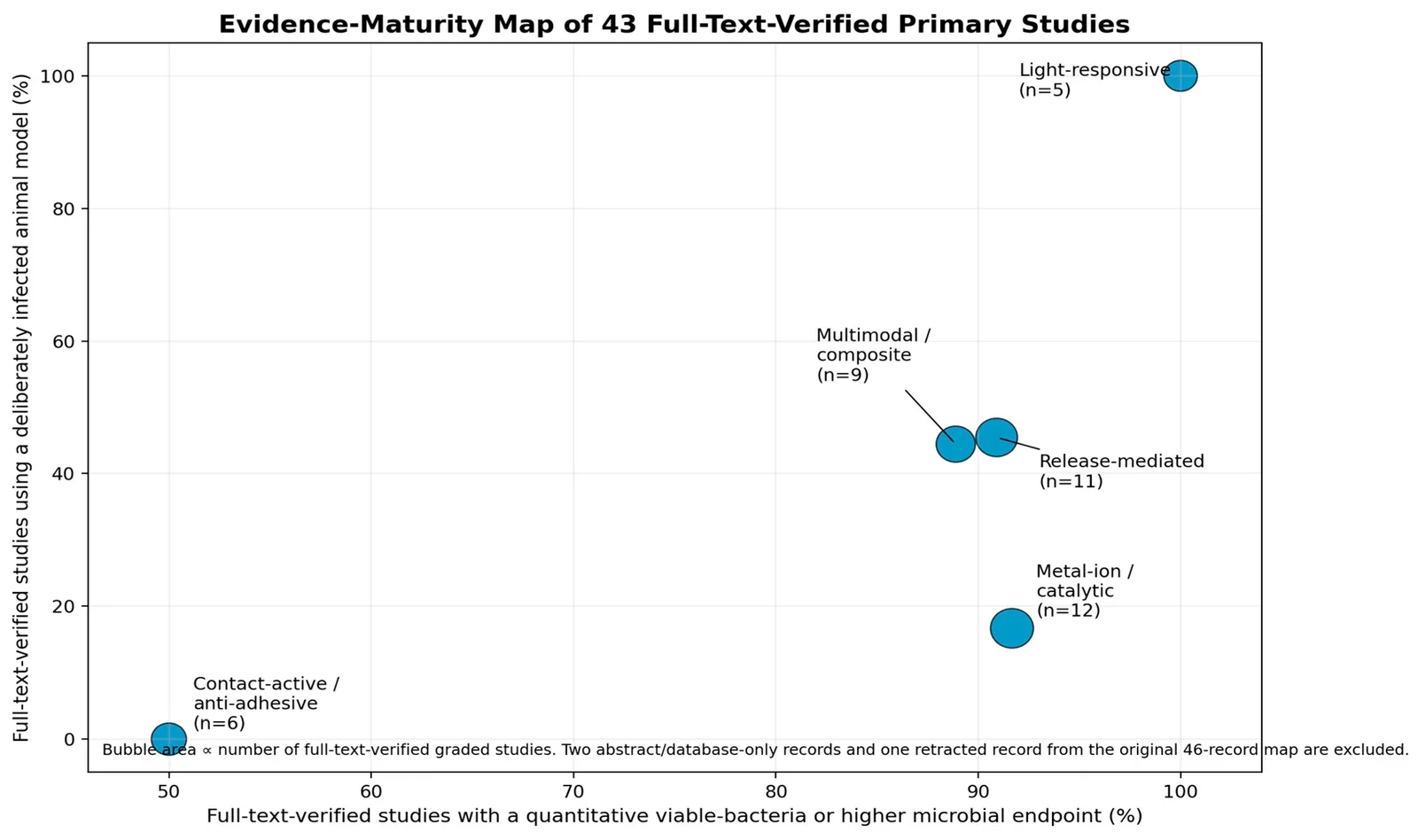
Quantitative evidence-maturity map for the 43 full-text-verified, non-retracted primary studies used for detailed grading. Bubble size represents the number of graded studies in each mechanism class; the *x*-axis shows the proportion with a quantitative viable-bacteria or higher microbial endpoint, and the *y*-axis shows the proportion using a deliberately infected animal model. Two abstract/database-only records, and one retracted record from the original 46-record map are excluded from these percentages. Mechanism-specific risks are compared in Table 2.

The most credible near-term environmental direction is a non-leaching, immobilized, regenerable antimicrobial interface. This prioritizes repeated pathogen log reduction and fouling resistance over single-use release. Figure 5 summarizes where the 43-study full-text-verified biomedical evidence currently sits by mechanism using two directly coded axes—quantitative microbial testing and deliberately infected animal deployment—while Table 2 separately lists unresolved translation risks.

## 7. Translation Bottlenecks, Standardization, and Biosafety

### 7.1. Sequential Translation-Gate Framework

Translation is evaluated as sequential gates rather than a single maturity level: T1, material identity and batch reproducibility; T2, quantitative antimicrobial exposure and efficacy; T3, mechanism-specific causality; T4, host selectivity; T5, validated sterilization; T6, storage stability; T7, resistance/adaptation and long-term material fate or leaching; and T8, large-animal, clinical, or environmental deployment. A formulation that reaches an infected animal model can still fail at T1, T3, or T5–T7. Across the reviewed corpus, the recurrent bottleneck is early transition from proof-of-concept efficacy to animal repair studies before exposure, causality, sterilization/storage, resistance, and fate have been frozen as engineering specifications.

### 7.2. Raw-Material, Batch, Sterilization, and Storage Control

T1 and T5 are coupled because SF molecular state depends on degumming, dissolution, dialysis, concentration, storage, and modification, while SS depends strongly on source and extraction severity [9,10,11,15,21]. Batch release should therefore include protein concentration, a molecular-weight distribution or validated proxy, secondary structure, gelation behavior, swelling, mechanical properties, degradation, and active-agent release. The intended sterilization process must then be applied to the final formulation, because irradiation, light exposure, oxidation, heat, or residual sterilants can alter network density, drug potency, and particle behavior [18,21,40].

Post-sterilization acceptance criteria should include gelation time, modulus, swelling, degradation, antimicrobial potency, release/activation profile, cytocompatibility, and the same microbial endpoint used for the efficacy claim. Accelerated and real-time storage, batch-to-batch reproducibility, and post-storage antimicrobial testing remain uncommon; consequently, preclinical efficacy should not be presented as shelf-life or manufacturability evidence.

### 7.3. Host Selectivity and Long-Term Fate

Antibacterial and host-toxic effects may share the same physical driver. Cationic charge, ROS, metal-ion release, nitric oxide, and heating can damage bacteria while also affecting keratinocytes, fibroblasts, endothelial cells, erythrocytes, or immune cells. Short-term viability and hemolysis assays are necessary but insufficient to define a therapeutic window. Longitudinal local inflammation, organ distribution, degradation products, metal retention, and repeated-exposure effects are especially important for nanoparticle-containing systems.

The available literature contains many short-term compatibility measurements but few integrated studies of antimicrobial exposure, host-cell selectivity, and material fate. This limitation is common to Ag, Ga, Zn, Se, MOF, nanozyme, and photoresponsive formulations [50,68,69,70,71,72,74,76,80].

### 7.4. Resistance and Microbial Adaptation

Localized delivery may reduce systemic exposure, but it does not remove evolutionary selection. Antibiotic release has the most obvious risk when a long release tail crosses sub-MIC concentrations. Metal systems can select altered uptake, efflux, sequestration, or oxidative-stress responses; cationic/contact-active interfaces can impose membrane-stress selection; and photothermal or photodynamic systems can repeatedly expose surviving populations to sublethal heat or ROS. Thus, ‘non-antibiotic’ and ‘resistance-proof’ are not interchangeable terms. The reviewed primary studies rarely included serial passage, post-exposure susceptibility, stable regrowth phenotyping, or genomic/transcriptional adaptation analysis.

Resistance assessment should include repeated passage at sublethal exposure, minimum inhibitory and bactericidal concentrations before and after exposure, biofilm regrowth, phenotype stability after drug-free recovery, and genomic or transcriptional analysis when adaptation is suspected.

### 7.5. Formal Claim Ceilings and Minimum Decisive Controls

The formal biological ceiling is C0–C5: C0, antimicrobial-related material functionality without a direct microbial effect; C1, growth inhibition; C2, quantitative viable-bacteria reduction; C3, viable reduction in a pre-established biofilm; C4, quantitative reduction of bacterial burden in deliberately infected tissue; and C5, durable or superior control versus an appropriate comparator with longitudinal/recurrence evidence. Translation gates T1–T8 are assessed separately. Mechanistic claims require controls tailored to the proposed mode of action: metal-ion systems require release measurements and ion-matched controls; ROS systems require scavenger or catalytic-inhibition rescue; photothermal systems require temperature-matched controls; antibiotic systems require exposure-matched free drug; anti-adhesive systems require separate adhesion and viability measurements; and multimodal systems require factorial component-ablation and interaction analysis. The mechanism-specific minimum controls, primary endpoints, and corresponding biological claim ceilings are summarized in Table 3.

## 8. Limitations

This evidence map has several methodological limitations. First, the review protocol was retrospective and was not prospectively registered, and raw discovery-hit counts were not archived. Second, PubMed/MEDLINE was the only formal bibliographic database used for retrospective re-verification; DOI-linked original-article sources and citation chaining improved source verification but do not substitute for prospective multi-database coverage. Third, no formal study-level risk-of-bias tool was applied because the corpus spans heterogeneous material, in vitro microbial, biofilm, and animal designs; the C0–C5 claim ceilings therefore describe the maximum endpoint directly demonstrated rather than a validated quality score. Fourth, no meta-analysis was undertaken by design because formulations, strains, inocula, hydrogel geometry, exposure time, and outcome units were too heterogeneous for defensible pooling.

Fifth, the C0–C5 biological claim ceiling, T1–T8 translation gates, and S0–S3 silk-contribution categories are author-generated analytical frameworks and have not been externally validated; they are intended to make assumptions explicit, not to function as established scoring instruments. Sixth, publication bias is likely to favor formulations reporting successful antibacterial or wound-healing outcomes, while null results, failed formulations, manufacturing failures, and negative long-term safety findings may be underrepresented. Finally, the second-round source audit identified one retracted record, which was excluded, and two records [87,88] that could not be verified beyond abstract/database level in the available audit trail and were therefore not used for detailed grading. Accordingly, descriptive proportions in this review characterize the retained, verifiable corpus and should not be interpreted as population-level effect estimates.

## 9. Future Perspectives

The next phase should prioritize quantitative source-to-function causality over additional compositional complexity. The field already contains many combinations of SF or SS with antibiotics, metals, nanoparticles, polyphenols, oxygen-generating agents, nanozymes, and photothermal materials. The decisive question is whether a defined silk molecular state or network architecture improves antimicrobial exposure, host selectivity, or durability relative to a simpler exposure-matched comparator.

Three priorities follow directly from the evidence map. First, source-to-function studies should vary a defined SF or SS molecular descriptor—such as molecular-weight distribution, β-sheet fraction, extraction severity, crosslink density, or mesh size—and link it to release/activation and quantitative killing. Second, biofilm and infected animal studies should separate anti-adhesion, formation inhibition, disruption, viable killing, and eradication, and should quantify tissue bacterial burden independently from wound repair. Third, translation should be gated: sterilization, storage, batch reproducibility, resistance/adaptation, and long-term fate should be passed before large-animal or clinical escalation.

A minimum decisive experiment for a new multifunctional formulation should compare the complete hydrogel with the silk network alone, each active component alone, all relevant partial combinations, and an exposure-matched free antimicrobial control. The primary endpoint should be selected in advance according to the intended claim: quantitative CFU for viable killing, a pre-established mature-biofilm assay for biofilm eradication, or quantitative tissue burden for infection clearance. Host repair and safety should be analyzed as independent outcomes.

For environmental translation, direct pathogen-control studies should precede claims based on adsorption or general material sustainability. The priority is not simply a biodegradable hydrogel, but a regenerable interface that maintains antimicrobial activity without unacceptable leaching, energy input, or ecotoxicity.

## 10. Conclusions

SF and SS provide complementary but non-equivalent material functions. SF most consistently contributes structural stability, β-sheet-dependent consolidation, processability, and transport control, whereas SS contributes hydrophilicity, ionizable and crosslinkable functional groups, and extraction-sensitive interfacial properties. Across the core evidence map, the direct bactericidal determinant is usually an incorporated antibiotic, metal/catalyst, cationic component, or photoactive phase rather than silk alone. Therefore, silk-specific antibacterial claims require matched component controls that demonstrate more than carrier or structural function.

The current evidence supports substantial in vitro antibacterial activity and a growing set of infected-wound models, but it does not yet justify broad claims of mature-biofilm eradication, reproducible quantitative infected-tissue clearance, mechanistic superiority, resistance prevention, manufacturing readiness, or environmental safety. The next advance in silk-derived antibacterial hydrogels is therefore unlikely to come primarily from adding another antimicrobial component. It will come from quantitatively linking silk molecular identity and network architecture to antimicrobial exposure, bacterial killing, host selectivity, sterilization/storage robustness, and long-term material fate.

## Figures and Tables

**Table 1:** Minimum material-reporting and evidence requirements for silk-derived antibacterial hydrogels.

Domain	Minimum Reporting Items	Interpretive Purpose	Common Overclaim Prevented
Silk identity	Silk species/source; SF versus SS content; residual sericin; protein concentration	Defines the actual biomaterial being compared	Treating all “SF” or “SS” preparations as chemically equivalent
Extraction and processing	Degumming/extraction conditions; dissolution; dialysis; storage; molecular-weight information	Links processing history to network and biological behavior	Attributing performance solely to the antimicrobial additive
Network formation	Crosslinker/initiator; substitution degree; gelation time; secondary structure; pore structure	Defines transport, stability, and active-group accessibility	Assuming identical release or contact activity across networks
Bulk properties	Swelling; degradation; rheology; compression/tension; adhesion	Connects material function with exposure and retention	Using mechanical improvement as evidence of antibacterial efficacy
Antimicrobial formulation	Active component; loading; spatial distribution; release or activation profile	Defines effective antimicrobial exposure	Reporting nominal loading without exposure data
Microbial assay	Strain; inoculum; medium; sample geometry; contact time; CFU/time-kill method	Enables comparison of antibacterial performance	Comparing percentage inhibition across incompatible assays
Biofilm assay	Biofilm age; biomass versus viable burden; imaging depth; regrowth	Distinguishes anti-adhesion from mature-biofilm eradication	Calling crystal-violet or adhesion data “biofilm clearance”
Host selectivity	Cell type; exposure route; hemolysis; irritation; tissue injury	Defines the antibacterial–host safety window	Inferring safety from a single short-term viability assay
Animal infection	Pathogen; inoculum; establishment period; tissue/swab CFU; sampling schedule	Separates infection control from wound repair	Using wound closure or inflammation as bacterial clearance
Manufacturing and fate	Sterilization; storage; batch reproducibility; leaching; resistance; long-term/ecotoxicity	Defines translation and environmental acceptability	Calling a laboratory formulation clinically or environmentally ready

**Table 2:** Quantitative cross-mechanism comparison of the 43 full-text-verified, non-retracted primary silk-derived antibacterial hydrogel studies used for detailed grading. The audit status of all 46 originally mapped core records is provided in Supplementary Table S1.

Mechanism	*n* (%)	Direct Antibacterial Determinant	Dominant SF/SS Role	Evidence Maturity and Infected Animal Deployment in the Graded Corpus	Main Translational/AMR Risk	Chronic-Wound Fit	Priority Decision and Decisive Control
Release-mediated	11 (25.6%)	Antibiotics or diffusible small molecules	Reservoir; diffusion, swelling, and degradation regulate exposure	10/11 reached quantitative viable-bacteria or higher endpoints. Deliberately infected animal models: 5/11 (45.5%). Quantitative infected-tissue burden remained inconsistent.	PK–PD mismatch, burst or prolonged sub-MIC exposure, batch-dependent release; highest conventional-antibiotic resistance-selection risk	High when release and tissue burden are explicitly linked	Prioritize exposure-matched free-agent controls, local release–kill linkage, and repeated susceptibility/passaging when antibiotics are used
Contact-active/anti-adhesive	6 (14.0%)	Cationic groups, chitosan derivatives, polyphenols or immobilized interfacial functions	Interfacial presentation, cohesion, wet stability and active-group accessibility	3/6 reached quantitative viable-bacteria or higher endpoints; evidence is strongest for adhesion prevention or surface-associated burden. Deliberately infected animal models: 0/6 (0%).	Protein/EPS fouling, loss of accessible charge/function, and detachment being misread as killing; adaptation risk lower than antibiotics but not zero	Moderate; potentially useful as a non-leaching adjunct	Separate attachment from viability; use standardized wash recovery, membrane-injury readouts, and pre-established biofilms for any anti-biofilm claim
Metal-ion/catalytic	12 (27.9%)	Ag, Ga^3+^, ZnO/MOFs, nanozymes or other ion/catalytic species	Ion/catalyst localization, release control and catalytic microenvironment	11/12 reached quantitative viable-bacteria or higher endpoints. Deliberately infected animal models: 2/12 (16.7%). Mechanism-specific rescue and quantitative tissue burden were sparse.	Host toxicity, leaching, oxidative injury, persistence and metal/oxidative adaptation or cross-resistance	Potentially high, but therapeutic window may be narrow	Require ion-release or catalytic-dose measurements plus ion-matched/chelation or ROS-scavenger rescue and long-term fate testing
Light-responsive	5 (11.6%)	Photothermal or photodynamic agents, often combined with Ag or nanomaterials	Photoactive-phase retention, hydration and local energy delivery	5/5 reached quantitative viable-bacteria or higher endpoints. Deliberately infected animal models: 5/5 (100%). Quantitative infected-tissue burden and matched thermal/ROS controls were heterogeneous.	Thermal injury, penetration limits, repeated-dose safety and nanoparticle persistence; lower antibiotic pressure but stress adaptation remains plausible	Conditional: strongest for accessible superficial wounds	Use temperature-matched inert heating or ROS-rescue controls, measured dosimetry, spatial/depth assessment and repeated-treatment safety
Multimodal/composite	9 (20.9%)	Multiple modules for bacterial control plus oxygenation, redox, immune, repair or monitoring functions	Spatial/temporal compartmentalization and co-localization of modules	8/9 reached quantitative viable-bacteria or higher endpoints. Deliberately infected animal models: 4/9 (44.4%). Preclinical outcomes are broad, but formal interaction/synergy testing is uncommon.	Causal ambiguity, manufacturing/sterilization complexity, module interactions and an expanded failure-mode space	Potentially high only when bacterial and host-repair endpoints are independently demonstrated	Do not add modules without factorial ablation; require independent bacterial, injury, inflammation and repair outcomes with interaction analysis

Notes: *n* values refer to the 43 full-text-verified, non-retracted studies used for detailed grading. Deliberately infected-animal-model counts are reported as *n*/ *N* so the percentages plotted in the corresponding percentages can be verified directly. Evidence maturity and translational risk are separate dimensions; use of an infected-animal model does not itself establish quantitative infected-tissue bacterial clearance or manufacturing readiness. PK–PD, pharmacokinetic–pharmacodynamic; MIC, minimum inhibitory concentration; AMR, antimicrobial resistance.

**Table 3:** Mechanism-specific minimum controls, primary endpoints, and claim ceilings.

Proposed Mechanism	Minimum Decisive Controls	Primary Endpoint	Maximum Biological Claim	Does not Establish
Released antibiotic or small molecule	Network alone; active agent alone; exposure-matched free agent; release profile	CFU/time-kill linked to local concentration	C2: localized antibacterial delivery under tested exposure	Resistance suppression or superiority to free drug without PK–PD comparison
Contact-active killing	Charge/function-matched inactive surface; wash controls; membrane-damage assay	Viable surface-associated bacteria plus membrane injury	C2: contact-associated killing at the tested interface	Anti-adhesion or mature-biofilm eradication
Anti-adhesion	Matched surface chemistry/roughness; standardized washing; viability after recovery	Attached viable bacteria and retention after washing	C1–C2: reduced bacterial attachment; viability claim only if measured	Bactericidal activity
Metal-ion killing	Ion-release profile; ion-matched soluble control; chelation/rescue where feasible	Viable bacteria versus released-ion exposure	C2: metal-associated viable-bacteria reduction; intracellular mechanism requires rescue/target evidence	Specific intracellular mechanism without rescue/target evidence
ROS or nanozyme catalysis	Catalyst-free control; substrate control; radical scavenger/catalytic inhibitor	CFU plus ROS and rescue	C2 (or C3 if pre-established biofilm): ROS-dependent contribution when rescue is demonstrated	Selective bacterial killing or long-term host safety
Photothermal action	Light-only; material-without-light; temperature-matched inert heating	CFU versus measured temperature/dose	C2 (or C4 if quantitative infected-tissue burden): photothermal contribution under defined dosimetry	Nanomaterial-specific synergy without temperature matching
Photodynamic action	Light-only; photosensitizer-only; oxygen/ROS measurement; scavenger control	CFU plus ROS-dependent reversal	C2 (or C3 if pre-established biofilm): photodynamic contribution with ROS-dependent reversal	Deep-tissue efficacy without penetration data
Multimodal synergy	All single components and partial combinations; exposure matching; interaction analysis	Independent bacterial, injury, inflammation, and repair outcomes	C2–C5 depending on independent bacterial endpoint and factorial interaction evidence	Mechanistic synergy from complete-versus-untreated comparison alone

## Data Availability

The study-level evidence summarized in this review is reported in the article and Supplementary Materials. The extended evidence matrix is available from the corresponding author upon reasonable request.

## Supplementary Materials

The following supporting information can be downloaded at: https://www.mdpi.com/article/10.3390/gels12090809/s1. Supplementary Methods; Figure S1, second-round evidence-set verification flow; Table S1, audit of the 46 originally mapped primary records, including full-text verification status, retraction exclusion, claim-ceiling coding, and silk-contribution coding [26,47,48,49,50,51,52,53,54,55,56,57,58,59,61,62,63,64,65,67,68,69,70,71,72,73,74,75,76,77,78,79,80,81,82,83,84,87,88,89,90,91,92,93,94].

## References

[1] Ghalei S., Handa H. (2022). A Review on Antibacterial Silk Fibroin-Based Biomaterials: Current State and Prospects. Mater. Today Chem..

[2] Zou P., Li X., Zhao H., Qu S., Jiang Z., Lei X., Wu C., Liu L. (2025). Recent advances of silk-fibroin-based hydrogel in the field of antibacterial application. Polym. Int..

[3] Li Z., Wang Y. (2026). Silk Fibroin as a Platform for Advanced Antibacterial Biomaterials: Structure, Immune Modulation, and Biomedical Applications. ACS Biomater. Sci. Eng..

[4] Zheng H., Zuo B. (2021). Functional silk fibroin hydrogels: Preparation, properties and applications. J. Mater. Chem. B.

[5] Wang J., Liu H., Shi X., Qin S., Liu J., Lv Q., Liu J., Li Q., Wang Z. (2024). Development and Application of an Advanced Biomedical Material—Silk Sericin. Adv. Mater..

[6] Wang S.L., Zhuo J.J., Fang S.M., Xu W., Yu Q.Y. (2024). Silk Sericin and Its Composite Materials with Antibacterial Properties to Enhance Wound Healing: A Review. Biomolecules.

[7] Sahoo J.K., Hasturk O., Falcucci T., Kaplan D.L. (2023). Silk chemistry and biomedical material designs. Nat. Rev. Chem..

[8] Zhang H., Xu D., Zhang Y., Li M., Chai R. (2022). Silk fibroin hydrogels for biomedical applications. Smart Med..

[9] Sun H., Sun Y., Li P., Lv Y., Zhang P. (2026). The structure and extraction of silk fibroin and its applications in fruit and vegetable preservation: A review. Food Chem. X.

[10] Jaramillo-Quiceno N., Callone E., Dirè S., Álvarez-López C., Motta A. (2021). Boosting sericin extraction through alternative silk sources. Polym. J..

[11] Zhu L., Lin J., Pei L., Luo Y., Li D., Huang Z. (2022). Recent Advances in Environmentally Friendly and Green Degumming Processes of Silk for Textile and Non-Textile Applications. Polymers.

[12] Sun Y., Ma L., Wei T., Zheng M., Mao C., Yang M., Shuai Y. (2024). Green, Low-carbon Silk-based Materials in Water Treatment: Current State and Future Trends. ChemSusChem.

[13] Yuan T., Li Z., Zhang Y., Shen K., Zhang X., Xie R., Liu F., Fan W. (2021). Injectable Ultrasonication-Induced Silk Fibroin Hydrogel for Cartilage Repair and Regeneration. Tissue Eng. Part A.

[14] Chen L., Sun L., Yao J., Zhao B., Shao Z., Chen X. (2022). Robust Silk Protein Hydrogels Made by a Facile One-Step Method and Their Multiple Applications. ACS Appl. Bio Mater..

[15] Liu J., Sun H., Peng Y., Chen L., Xu W., Shao R. (2022). Preparation and Characterization of Natural Silk Fibroin Hydrogel for Protein Drug Delivery. Molecules.

[16] Wu J., Lei J., Chen M., Sun Y., Hou J., Li S., Liu G., Zhang M., Shen Y., Zhang F. (2023). Synthesis and Characterization of Photo-Cross-Linkable Silk Fibroin Methacryloyl Hydrogel for Biomedical Applications. ACS Omega.

[17] Wang R., He X., Chen Z., Su S., Bai J., Liu H., Zhou F. (2024). A nanoparticle reinforced microporous methacrylated silk fibroin hydrogel to promote bone regeneration. Biomater. Sci..

[18] Thongnuek P., Kanokpanont S., Uttayarat P., Damrongsakkul S. (2023). Hydrogelation of Regenerated Silk Fibroin via Gamma Irradiation. Polymers.

[19] Baptista-Silva S., Bernardes B.G., Borges S., Rodrigues I., Fernandes R., Gomes-Guerreiro S., Pinto M.T., Pintado M., Soares R., Costa R. (2022). Exploring Silk Sericin for Diabetic Wounds: An In Situ-Forming Hydrogel to Protect against Oxidative Stress and Improve Tissue Healing and Regeneration. Biomolecules.

[20] Baptista-Silva S., Borges S., Costa-Pinto A.R., Costa R., Amorim M., Dias J.R., Ramos Ó., Alves P., Granja P.L., Soares R. (2021). In Situ Forming Silk Sericin-Based Hydrogel: A Novel Wound Healing Biomaterial. ACS Biomater. Sci. Eng..

[21] Zhang Y., Wang S., Li Y., Li X., Du Z., Liu S., Song Y., Li Y., Zhang G. (2023). A Sterile, Injectable, and Robust Sericin Hydrogel Prepared by Degraded Sericin. Gels.

[22] Zhang Y., Wu T., Shen C., Xu G., Chen H., Yan H., Xiong M., Zhang G. (2022). A Robust Sericin Hydrogel Formed by a Native Sericin from Silkworm Bodies. Fibers Polym..

[23] Veiga A., Ribeiro V., Ramírez-Jiménez R.A., Aguilar M.R., Rojo L., Oliveira A.L. (2025). Biofunctional silk sericin hydrogels: A versatile platform with potential for tissue healing and regeneration. Colloids Surf. B Biointerfaces.

[24] Arango M.C., Jaramillo-Quiceno N., Badia J.D., Cháfer A., Cerisuelo J.P., Álvarez-López C. (2024). The Impact of Green Physical Crosslinking Methods on the Development of Sericin-Based Biohydrogels for Wound Healing. Biomimetics.

[25] Ziadlou R., Rotman S., Teuschl A., Salzer E., Barbero A., Martin I., Alini M., Eglin D., Grad S. (2021). Optimization of hyaluronic acid-tyramine/silk-fibroin composite hydrogels for cartilage tissue engineering and delivery of anti-inflammatory and anabolic drugs. Mater. Sci. Eng. C Mater. Biol. Appl..

[26] Ke X., Dong Z., Tang S., Chu W., Zheng X., Zhen L., Chen X., Ding C., Luo J., Li J. (2020). A natural polymer based bioadhesive with self-healing behavior and improved antibacterial properties. Biomater. Sci..

[27] Li Y., Wang S., Li Y., Zhang G., Wu T., Wei Y., Cao X., Yan H., Liang P., Yan Z. (2023). Resveratrol loaded native silk fiber-sericin hydrogel double interpenetrating bioactive wound dressing facilitates full-thickness skin wound healing. Biomed. Mater..

[28] Wang J., Zhang N., Tan Y., Fu F., Liu G., Fang Y., Zhang X.X., Liu M., Cheng Y., Yu J. (2022). Sweat-Resistant Silk Fibroin-Based Double Network Hydrogel Adhesives. ACS Appl. Mater. Interfaces.

[29] Liu X., Yang Y., Yu H., Wang L., Sheng Y., Huang Z., Yang J., Ni Z., Shen D. (2022). Instant and Tough Adhesives for Rapid Gastric Perforation and Traumatic Pneumothorax Sealing. Adv. Healthc. Mater..

[30] Lu Y., Huang X., Luo Y., Zhu R., Zheng M., Yang J., Bai S. (2023). Silk Fibroin-Based Tough Hydrogels with Strong Underwater Adhesion for Fast Hemostasis and Wound Sealing. Biomacromolecules.

[31] Piluso S., Flores Gomez D., Dokter I., Moreira Teixeira L., Li Y., Leijten J., van Weeren R., Vermonden T., Karperien M., Malda J. (2020). Rapid and cytocompatible cell-laden silk hydrogel formation via riboflavin-mediated crosslinking. J. Mater. Chem. B.

[32] Shi X., Wang X., Shen W., Yue W. (2023). Biocompatibility of silk methacrylate/gelatin-methacryloyl composite hydrogel and its feasibility as a vascular tissue engineering scaffold. Biochem. Biophys. Res. Commun..

[33] Jian G., Li D., Ying Q., Chen X., Zhai Q., Wang S., Mei L., Cannon R.D., Ji P., Liu W. (2023). Dual Photo-Enhanced Interpenetrating Network Hydrogel with Biophysical and Biochemical Signals for Infected Bone Defect Healing. Adv. Healthc. Mater..

[34] Hong H., Seo Y.B., Kim D.Y., Lee J.S., Lee Y.J., Lee H., Ajiteru O., Sultan M.T., Lee O.J., Kim S.H. (2020). Digital light processing 3D printed silk fibroin hydrogel for cartilage tissue engineering. Biomaterials.

[35] Xu L., Zhang Z., Jorgensen A.M., Yang Y., Jin Q., Zhang G., Cao G., Fu Y., Zhao W., Ju J. (2023). Bioprinting a skin patch with dual-crosslinked gelatin (GelMA) and silk fibroin (SilMA): An approach to accelerating cutaneous wound healing. Mater. Today Bio.

[36] Li Y., Zhang J., Wang C., Jiang Z., Lai K., Wang Y., Yang G. (2023). Porous composite hydrogels with improved MSC survival for robust epithelial sealing around implants and M2 macrophage polarization. Acta Biomater..

[37] Shibata M., Okahisa Y. (2024). Tough gelatine hydrogels reinforced with silk fibroin nanofiber. Heliyon.

[38] Wu W., Dong Y., Liu H., Jiang X., Yang L., Luo J., Hu Y., Gou M. (2023). 3D printed elastic hydrogel conduits with 7,8-dihydroxyflavone release for peripheral nerve repair. Mater. Today Bio.

[39] Cai Y., Huang Q., Wang P., Ye K., Zhao Z., Chen H., Liu Z., Liu H., Wong H., Tamtaji M. (2022). Conductive Hydrogel Conduits with Growth Factor Gradients for Peripheral Nerve Repair in Diabetics with Non-Suture Tape. Adv. Healthc. Mater..

[40] Tutar R., Yüce-Erarslan E., İzbudak B., Bal-Öztürk A. (2022). Photocurable silk fibroin-based tissue sealants with enhanced adhesive properties for the treatment of corneal perforations. J. Mater. Chem. B.

[41] Liu Y., Zhang Z., Zhang Y., Luo B., Liu X., Cao Y., Pei R. (2023). Construction of adhesive and bioactive silk fibroin hydrogel for treatment of spinal cord injury. Acta Biomater..

[42] Lu X., Guo H., Li J., Sun T., Xiong M. (2021). Recombinant Human Bone Morphogenic Protein-2 Immobilized Fabrication of Magnesium Functionalized Injectable Hydrogels for Controlled-Delivery and Osteogenic Differentiation of Rat Bone Marrow-Derived Mesenchymal Stem Cells in Femoral Head Necrosis Repair. Front. Cell Dev. Biol..

[43] Rodrigues C.L., Tomoda B.T., Viganó J., Braga A.R.C., de Moraes M.A., Veggi P.C. (2024). Production and Characterization of Silk Fibroin–Aloe vera Hydrogel: A Study on Extraction, Hydrogel Properties, and Release Mechanism. ACS Omega.

[44] Jaramillo-Quiceno N., Rueda-Mira S., Santa Marín J.F., Álvarez-López C. (2023). Development of a novel silk sericin-based hydrogel film by mixture design. J. Polym. Res..

[45] Du P., Diao L., Lu Y., Liu C., Li J., Chen Y., Chen J., Lv G., Chen X. (2023). Heparin-based sericin hydrogel-encapsulated basic fibroblast growth factor for in vitro and in vivo skin repair. Heliyon.

[46] Bai L., Zhang X., Li X., Wang S., Zhang Y., Xu G. (2023). Impact of a Novel Hydrogel with Injectable Platelet-Rich Fibrin in Diabetic Wound Healing. J. Diabetes Res..

[47] Tao G., Cai R., Wang Y., Zuo H., He H. (2021). Fabrication of antibacterial sericin based hydrogel as an injectable and mouldable wound dressing. Mater. Sci. Eng. C Mater. Biol. Appl..

[48] Mei J., Zhou J., Kong L., Dai Y., Zhang X., Song W., Zhu C. (2022). An injectable photo-cross-linking silk hydrogel system augments diabetic wound healing in orthopaedic surgery through spatiotemporal immunomodulation. J. Nanobiotechnol..

[49] Zhu Y., Liu H., Qin S., Yang C., Lv Q., Wang Z., Wang L. (2022). Antibacterial Sericin Cryogels Promote Hemostasis by Facilitating the Activation of Coagulation Pathway and Platelets. Adv. Healthc. Mater..

[50] Laomeephol C., Punjataewakupt A., Kanchanasin P., Phongsopitanun W., Ferreira H., Neves N.M., Aramwit P. (2025). Silver Cross-Linking of Silk Sericin-Based Hydrogels for Improved Stability and Broad-Spectrum Antimicrobial Properties. ACS Appl. Bio Mater..

[51] Liu Y., Fan J., Lv M., She K., Sun J., Lu Q., Han C., Ding S., Zhao S., Wang G. (2021). Photocrosslinking silver nanoparticles-aloe vera-silk fibroin composite hydrogel for treatment of full-thickness cutaneous wounds. Regen. Biomater..

[52] Bakadia B.M., Boni B.O.O., Ahmed A.A.Q., Zheng R., Shi Z., Ullah M.W., Lamboni L., Yang G. (2022). In Situ Synthesized Porous Bacterial Cellulose/Poly(vinyl alcohol)-Based Silk Sericin and Azithromycin Release System for Treating Chronic Wound Biofilm. Macromol. Biosci..

[53] Bakadia B.M., Lamboni L., Ahmed A.A.Q., Zheng R., Boni B.O.O., Shi Z., Song S., Souho T., Mukole B.M., Qi F. (2023). Antibacterial silk sericin/poly(vinyl alcohol) hydrogel with antifungal property for potential infected large burn wound healing: Systemic evaluation. Smart Mater. Med..

[54] Li Y., Wei Y., Gao Z., Zhang G., Zhu M., Yan H., Zhang Y. (2025). A multifunctional sericin hydrogel system fabricated via one-step acetic acid induction for wound management. J. Mater. Chem. B.

[55] Yin C., Han X., Lu Q., Qi X., Guo C., Wu X. (2022). Rhein incorporated silk fibroin hydrogels with antibacterial and anti-inflammatory efficacy to promote healing of bacteria-infected burn wounds. Int. J. Biol. Macromol..

[56] Jafarbeglou M., Meimandi-Parizi A., Derakhshandeh A., Khodakaram-Tafti A., Bigham-Sadegh A., Arkan P., Jafarbeglou M. (2024). Silk fibroin/chitosan thiourea hydrogel scaffold with vancomycin and quercetin-loaded PLGA nanoparticles for treating chronic MRSA osteomyelitis in rats. Int. J. Pharm..

[57] Phewchan P., Laoruengthana A., Chomchalao P., Lamlertthon S., Tiyaboonchai W. (2024). Vancomycin-Loaded Silk Fibroin/Calcium Phosphate/Methylcellulose-Based In Situ Thermosensitive Hydrogel: A Potential Function for Bone Regeneration. Gels.

[58] Watchararot T., Soomherun N., Jitpibull J., Keeratihattayakorn S., Ratanavaraporn J. (2025). Antibacterial, angiogenic and irritation evaluation of mupirocin-loaded silk fibroin hydrogel. J. Drug Deliv. Sci. Technol..

[59] Mao Y., Zhang Y., Wang Y., Zhou T., Ma B., Zhou P. (2023). A multifunctional nanocomposite hydrogel with controllable release behavior enhances bone regeneration. Regen. Biomater..

[60] Godiya C.B., Cheng X., Deng G., Li D., Lu X. (2019). Silk fibroin/polyethylenimine functional hydrogel for metal ion adsorption and upcycling utilization. J. Environ. Chem. Eng..

[61] Xu Z., Chen T., Zhang K., Meng K., Zhao H. (2021). Silk fibroin/chitosan hydrogel with antibacterial, hemostatic and sustained drug-release activities. Polym. Int..

[62] Zhang L., Zhang Y., Ma F., Liu X., Liu Y., Cao Y., Pei R. (2022). A low-swelling and toughened adhesive hydrogel with anti-microbial and hemostatic capacities for wound healing. J. Mater. Chem. B.

[63] Khodaei A., Johari N., Jahanmard F., Cecotto L., Khosravimelal S., Madaah Hosseini H.R., Bagheri R., Samadikuchaksaraei A., Amin Yavari S. (2024). Particulate 3D Hydrogels of Silk Fibroin-Pluronic to Deliver Curcumin for Infection-Free Wound Healing. Biomimetics.

[64] Chen W., Xiao X., Qian P., Zhou Z., Ruan W. (2025). Antibiotic-free silk sericin hydrogel with antibacterial and antioxidative dual functionalities promotes wound healing. Int. J. Biol. Macromol..

[65] Wu Y., Chen J., Han L., Zhang Y., Wei L. (2026). Multifunctional Silk Fibroin Hydrogel with Antibacterial and Regenerative Properties for Accelerated Wound Healing. Gels.

[66] Tuwalska A., Grabska-Zielińska S., Sionkowska A. (2022). Chitosan/Silk Fibroin Materials for Biomedical Applications—A Review. Polymers.

[67] El-Samad L.M., Hassan M.A., Basha A.A., El-Ashram S., Radwan E.H., Abdul Aziz K.K., Tamer T.M., Augustyniak M., El Wakil A. (2022). Carboxymethyl cellulose/sericin-based hydrogels with intrinsic antibacterial, antioxidant, and anti-inflammatory properties promote re-epithelization of diabetic wounds in rats. Int. J. Pharm..

[68] Zheng H., Huang Z., Chen T., Sun Y., Chen S., Bu G., Guan H. (2022). Gallium ions incorporated silk fibroin hydrogel with antibacterial efficacy for promoting healing of Pseudomonas aeruginosa-infected wound. Front. Chem..

[69] Zhu Z., Liu Y., Chen J., He Z., Tan P., He Y., Pei X., Wang J., Tan L., Wan Q. (2022). Structural-Functional Pluralistic Modification of Silk Fibroin via MOF Bridging for Advanced Wound Care. Adv. Sci..

[70] Yang C.M., Lee J., Lee S.Y., Lee H., Chathuranga K., Lee J., Park W. (2022). Silk Fibroin/Tannin/ZnO Nanocomposite Hydrogel with Hemostatic Activities. Gels.

[71] Li K., Yan X., Du Y., Chen S., You Y., Wu W., Liu X., Dong L., Wang Q., Wang Q. (2023). Silk Fibroin Nanozyme Hydrogel with Self-Supplied H_2_O_2_ for Enhanced Antibacterial Therapy. ACS Appl. Nano Mater..

[72] Yang D., Zhao W., Zhang S., Liu Y., Teng J., Ma Y., Huang R., Wei H., Chen H., Zhang J. (2024). Dual Self-Assembly of Puerarin and Silk Fibroin into Supramolecular Nanofibrillar Hydrogel for Infected Wound Treatment. Adv. Healthc. Mater..

[73] Lalebeigi F., Kashtiaray A., Aghamirza Moghim Aliabadi H., Moghadaskhou F., Pajoum Z., Nokandeh S.M., Mahdavi M., Eivazzadeh-Keihan R., Maleki A. (2024). Agar-tragacanth/silk fibroin hydrogel containing Zn-based MOF as a novel nanobiocomposite with biological activity. Sci. Rep..

[74] Wiita E.G., Toprakcioglu Z., Jayaram A.K., Knowles T.P.J. (2024). Selenium-silk microgels as antifungal and antibacterial agents. Nanoscale Horiz..

[75] Li Y., Yong D., Shen J., Bian R., Wang Y. (2025). Silver nanoparticle-loaded konjac glucomannan/silk fibroin composite hydrogels for enhanced wound healing. Int. J. Biol. Macromol..

[76] Yao C., Wu Q., Zhao Y., Li H., He J., Liu L., Huang Y., Cheng F. (2025). Engineered Au@MOFs silk fibroin-based hydrogel phototherapy platform for enhanced wound healing performance. Int. J. Biol. Macromol..

[77] Zhang M., Wang D., Ji N., Lee S., Wang G., Zheng Y., Zhang X., Yang L., Qin Z., Yang Y. (2021). Bioinspired Design of Sericin/Chitosan/Ag@MOF/GO Hydrogels for Efficiently Combating Resistant Bacteria, Rapid Hemostasis, and Wound Healing. Polymers.

[78] Yan S., Li J., Gao Y., You J., Xu S., Wang C., Yang Y., Wu X. (2023). Encapsulation of Sericin-Decorated Efficient Agents in Silk Hydrogels for Wound Dressings. ACS Appl. Mater. Interfaces..

[79] Cheng Q., He Y., Ma L., Lu L., Cai J., Xu Z., Shuai Y., Wan Q., Wang J., Mao C. (2024). Regenerated silk fibroin coating stable liquid metal nanoparticles enhance photothermal antimicrobial activity of hydrogel for wound infection repair. Int. J. Biol. Macromol..

[80] Yao C., Zhao Y., Wu Y., Wang H., Li H., He J., Liu L., Huang Y., Cheng F. (2026). Single-Doped W_18_O_49−x_@Au-Embedded Dual-Network Silk Fibroin Hydrogel for NIR-Responsive Phototherapeutic Wound Healing. Adv. Healthc. Mater..

[81] Yan S., Yu Z., Yang H., Yang Y., Qin J., Wu X., Lin G. (2025). Near-infrared light-triggered silk fibroin hydrogels integrated with polydopamine-modified nanoparticles for enhanced wound healing and infection control. Int. J. Biol. Macromol..

[82] Chu W., Wang P., Ma Z., Peng L., Guo C., Fu Y., Ding L. (2023). Lupeol-loaded chitosan-Ag^+^ nanoparticle/sericin hydrogel accelerates wound healing and effectively inhibits bacterial infection. Int. J. Biol. Macromol..

[83] Wang X., Wang S., Tehoungue A., Li Y., Li X., Yanhui Zhu M., Zhang G., Zhang Y. (2025). A robust visualized sericin hydrogel dressing with excellent antioxidative and antimicrobial activities facilitates diabetic wound healing. J. Mech. Behav. Biomed. Mater..

[84] Han J., Meng Q., Xue S., Su W., Wu J. (2025). Silk fibroin methacryloyl hydrogel loaded with silver-gallic acid nanoparticles for enhanced diabetic wound healing. Int. J. Biol. Macromol..

[85] Napavichayanun S., Yamdech R., Pienpinijtham P., Srichana T., Chencharoenwong S., Reddy N., Aramwit P. (2022). Using polyvinyl alcohol-ionic hydrogels containing a wound healing agent to manage wounds in different environments. J. Wound Care.

[86] Yang C., Li S., Huang X., Chen X., Shan H., Chen X., Tao L., Zhang M. (2022). Silk Fibroin Hydrogels Could Be Therapeutic Biomaterials for Neurological Diseases. Oxid. Med. Cell Longev..

[87] Schnaider L., Toprakcioglu Z., Ezra A., Liu X., Bychenko D., Levin A., Gazit E., Knowles T.P.J. (2020). Biocompatible Hybrid Organic/Inorganic Microhydrogels Promote Bacterial Adherence and Eradication In Vitro and In Vivo. Nano Lett..

[88] Eivazzadeh-Keihan R., Khalili F., Aghamirza Moghim Aliabadi H., Maleki A., Madanchi H., Ziaei Ziabari E., Salimi Bani M. (2020). Alginate hydrogel-poly(vinyl alcohol)/silk fibroin/magnesium hydroxide nanorods: A novel scaffold with biological and antibacterial activity and improved mechanical properties. Int. J. Biol. Macromol..

[89] Dong M., Mao Y., Zhao Z., Zhang J., Zhu L., Chen L., Cao L. (2022). Novel fabrication of antibiotic containing multifunctional silk fibroin injectable hydrogel dressing to enhance bactericidal action and wound healing efficiency on burn wound: In vitro and in vivo evaluations. Int. Wound J..

[90] Sun M., Zhong X., Dai M., Feng X., Tang C., Cao L., Liu L. (2024). Antibacterial microneedle patch releases oxygen to enhance diabetic wound healing. Mater. Today Bio.

[91] Yan S., Xu S., Wang Y., You J., Guo C., Wu X. (2024). A Hydrogel Dressing Comprised of Silk Fibroin, Ag Nanoparticles, and Reduced Graphene Oxide for NIR Photothermal-Enhanced Antibacterial Efficiency and Skin Regeneration. Adv. Healthc. Mater..

[92] Guo Z., Yan L., Zhou B., Zhao P., Wang W., Dong S., Cheng B., Yang J., Wang X., Li B. (2023). In situ photo-crosslinking silk fibroin based hydrogel accelerates diabetic wound healing through antibacterial and antioxidant. Int. J. Biol. Macromol..

[93] Zeng Z., Guo J., Shen G., Guo C., Pei D., Lu D., Geng Z., Huang J., Yu S. (2023). Antibacterial-Antioxidative Thiolated Gelatin/Methacrylated Silk Fibroin Hydrogels with Nitric Oxide Release Catalyzed by Metal-Polyphenol Nanoparticles for MRSA-Infected Wound Healing. Biomacromolecules.

[94] Jing Y., Ruan L., Jiang G., Nie L., Shavandi A., Sun Y., Xu J., Shao X., Zhu J. (2023). Regenerated silk fibroin and alginate composite hydrogel dressings loaded with curcumin nanoparticles for bacterial-infected wound closure. Biomater. Adv..

