# Supplementary material for: Silk-Derived Antibacterial Hydrogels: Material Identity, Mechanistic Evidence, and Translation
Source: Gels. 2026 Sep 3;12(9):809. doi: 10.3390/gels12090809 (PMC13606779; doi:10.3390/gels12090809)
Supplement: Supplementary file 1 [file gels-12-00809-s001.zip › gels-4514937-supplementary.pdf]

Supplementary Materials

Citation numbers refer to the reference list in the main manuscript.

Supplementary Methods: Search Verification and Evidence Coding

Review definition and scope. The revised article is a structured evidence-mapping and critical synthesis of a frozen contemporary corpus rather than a prospectively registered systematic review. The graded quantitative core window was January 2020 through July 2026, selected to capture current photocrosslinkable, catalytic, nano-enabled, light-responsive, and multimodal silk-derived antibacterial hydrogel architectures. Foundational pre-2020 studies may be used for molecular or materials context but are not counted in the graded quantitative map.

Evidence-set verification. The frozen corpus contains 95 references: 46 primary records originally mapped to the antibacterial core, 35 supporting primary silk-derived hydrogel studies, and 14 silk-specific reviews or methodological sources. PubMed/MEDLINE was the sole formal bibliographic database used for retrospective re-verification, supplemented by DOI-linked original-article sources and backward citation chaining. A second database was not added after the evidence set had been frozen because doing so without re-running the full discovery-to-eligibility workflow would create a hybrid process that could misleadingly imply prospective multi-database coverage. This is treated as a limitation in the main manuscript. During the second-round audit, all 46 original core records were re-opened at the original-source level: 43 were full-text verified and retained for detailed grading, two [74,83] could be verified only at abstract/database level and were retained descriptively but not graded, and one [61] was found to have been formally retracted and was excluded from all quantitative synthesis and grading. The initial exploratory search was not prospectively registered and raw discovery-hit counts were not archived; Figure S1 therefore documents the auditable verification flow rather than invented PRISMA identification numbers.

Reproducible verification query. The core Boolean concept block was: ("silk fibroin" OR sericin OR "silk protein" OR SilkMA OR SilMA) AND (hydrogel OR cryogel OR injectable OR photocrosslink* OR "photo-crosslink*" OR "double network" OR "3D print*") AND (antibacter* OR antimicrobial* OR biofilm OR "infected wound" OR MRSA OR "wound infection"). A contextual environmental block added ("water treatment" OR disinfection OR antifouling OR remediation OR regenerable OR reusable). Search terms were adapted to the syntax of each retrieval interface, and citation chaining was used to identify silk-specific material studies not captured by the antibacterial block.

Eligibility. A study entered the 46-study core map when SF or SS was a load-bearing network component, critical crosslinking component, or indispensable structural phase of the final hydrogel and at least one direct microbial endpoint was reported. Studies in which sericin served only as a nanoparticle template or silk was absent from the final load-bearing network were excluded from the antibacterial core. Supporting primary studies were retained separately when they informed material identity, crosslinking, mechanics, degradation, transport, sterilization, repair, or sustainability.

Orthogonal coding. Biological claim ceilings are C0, antimicrobial-related material functionality without a direct microbial effect; C1, growth inhibition; C2, quantitative reduction in viable bacteria; C3, reduction in a pre-established viable biofilm; C4, quantitative reduction of bacterial burden in deliberately infected tissue; and C5, durable or superior control versus an appropriate comparator with longitudinal/recurrence evidence. Translation maturity is assessed independently by gates T1–T8 (material identity/batch reproducibility, quantitative efficacy, mechanistic causality, host selectivity, sterilization, storage, resistance/adaptation and long-term fate, and deployment). Silk contribution is coded independently as S0, additive-dominated matrix role; S1, measured silk-assisted structural/release/interfacial contribution; S2, silk-specific antimicrobial contribution supported by matched component controls; and S3, causal intrinsic silk-dependent killing. No study is promoted from S0/S1 to S2/S3 solely because SF or SS is present in the formulation. Detailed C0–C5 and S0–S3 coding is applied only to full-text-verified, non-retracted studies; abstract/database-only records are explicitly marked NG (not graded).

Biofilm terminology. Anti-adhesion, inhibition of biofilm formation, disruption of an established biofilm, killing within an established biofilm, and eradication are treated as non-equivalent claims. “Eradication” requires a below-detection viable endpoint together with absent regrowth after material removal. Wound closure, inflammatory markers, and histologic repair are coded separately from pathogen burden.

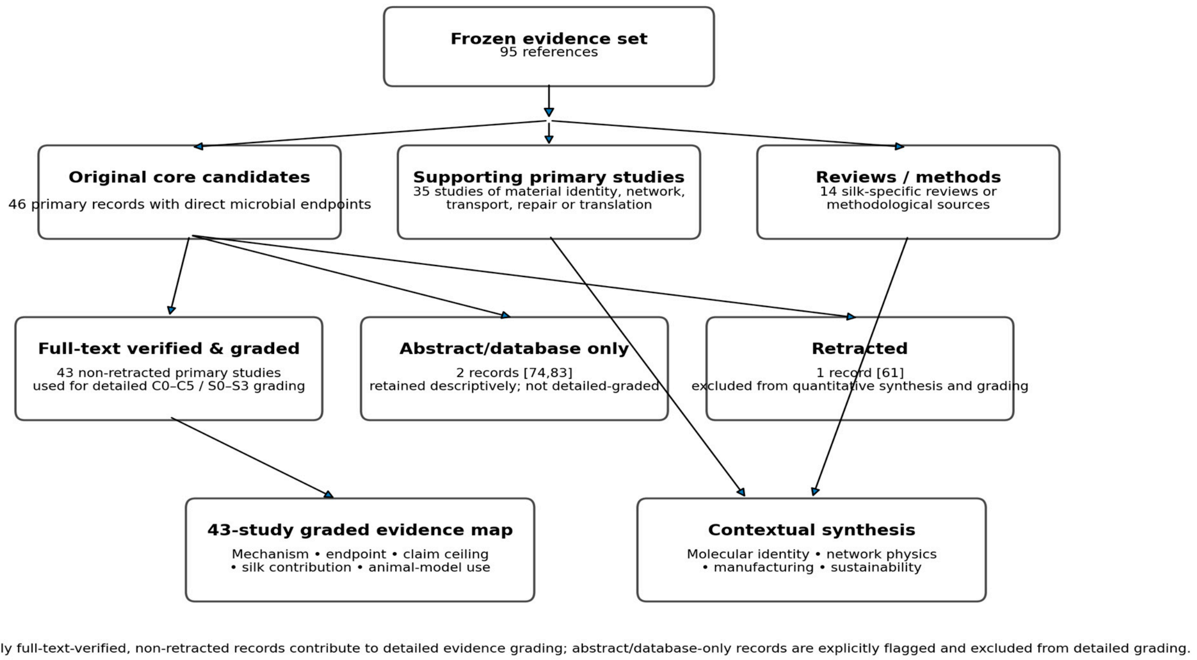

Figure S1. Second-round evidence-set verification flow. The frozen corpus contains 95 references. Of 46 primary records originally mapped to the antibacterial core, 43 were full-text verified and retained for detailed grading, two [74,83] were available only at abstract/database level and were not detailed-graded, and one [61] was retracted and excluded from quantitative synthesis. Raw discovery-hit counts from the original exploratory phase were not prospectively archived and are therefore not retrospectively invented.

Quantitative graded-study distribution. Among the 43 full-text-verified, non-retracted primary studies used for detailed grading, metal-ion/nanozyme/catalytic systems accounted for 12/43 (27.9%), release-mediated systems 11/43 (25.6%), multimodal systems 9/43 (20.9%), contact-active/anti-adhesive systems 6/43 (14.0%), and light-responsive systems 5/43 (11.6%). The study-level audit below retains all 46 originally mapped records for transparency. NR denotes 'not available as a harmonizable numeric value in the present extraction,' whereas NG denotes 'not graded because full-text verification was insufficient or the record was excluded.'

Table S1. Audit of the 46 primary records originally mapped to the antibacterial core. Detailed claim-ceiling and silk-contribution grading is based on 43 full-text-verified, non-retracted studies; abstract/database-only records are marked NG and the retracted record is excluded.

Mechanism	Reference	Composition	Structural SF/SS role	Direct antibacterial determinant	Microorganism(s)	Standardized microbial endpoint	Animal infection evidence	Claim ceiling	Silk contribution	Verification status; selected quantitative descriptor / NR
Release-mediated	Mei et al., 2022 [48]	Sil-MA/AgNP/metformin-microsphere hydrogel	Sil-MA is the photocrosslinked matrix	Ag nanoparticles, metformin-containing microspheres	Staphylococcus aureus and Escherichia coli	Quantitative viable-bacteria reduction in vitro (C2).	Diabetic wound-repair model without deliberate bacterial inoculation; antibacterial claim based on in vitro CFU.	C2	S1: measured/functional silk-assisted structural, release, stabilization, or interfacial role; direct silk-dependent killing not established.	FULL-TEXT VERIFIED. In vitro log10 CFU: S. aureus 7.72±0.10→6.90±0.09; E. coli 7.15±0.09→6.30±0.43 (Sil-MA control→staged Ag/metformin formulation).
Release-mediated	Liu et al., 2021 [51]	Photocross-linked SF/AgNP/Aloe vera	SF is the photo-crosslinked matrix	Ag nanoparticles and Aloe vera	Escherichia coli and Staphylococcus aureus.	Quantitative viable-bacteria reduction in vitro (C2).	Full-thickness wound model without a deliberately established infection; antibacterial claim based on in vitro testing.	C2	S0: antimicrobial effect is additive-dominated; SF/SS mainly provides matrix, carrier, or co-network function.	FULL-TEXT VERIFIED. NR in harmonized quantitative extraction; endpoint category and major limitation retained for cross-study comparison.
Release-mediated	Dong M et al., 2022 [52]	SF/graphene oxide/PEG/ciprofloxacin	SF is the principal injectable network	Ciprofloxacin, graphene oxide is a reinforcing/adsorptive component.	S. aureus and E. coli in Methods; S. aureus and P. aeruginosa in figures/results; internal strain-reporting inconsistency retained as a study limitation.	Growth inhibition / zone / OD or another non-CFU endpoint (C1).	Non-inoculated deep-burn model; no quantitative infected-tissue burden.	C1	S0: antimicrobial effect is additive-dominated; SF/SS mainly provides matrix, carrier, or co-network function.	FULL-TEXT VERIFIED. NR in harmonized quantitative extraction; endpoint category and major limitation retained for cross-study comparison.
Release-mediated	Bakadia et al., 2022 [53]	Bacterial cellulose/PVA/sericin/azithromycin	Sericin is a functional hydrogel component	Azithromycin, sericin-associated bioactivity	Chronic-wound biofilm organisms; strain identifiers NR	Pre-established biofilm inhibition/disruption; C3. Eradication below detection/no regrowth was not claimed.	Wound model reported; claim ceiling is based on the pre-established biofilm experiment rather than wound closure.	C3	S1: measured/functional silk-assisted structural, release, stabilization, or interfacial role; direct silk-dependent killing not established.	FULL-TEXT VERIFIED. Porosity 53.5±2.3%→83.5±2.9%; pore size 2.3±1.9→16.8±4.5 μm; fiber diameter 35.5±10→120±27.4 nm; biofilm inhibition/disruption assessed.

Mechanism	Reference	Composition	Structural SF/SS role	Direct antibacterial determinant	Microorganism(s)	Standardized microbial endpoint	Animal infection evidence	Claim ceiling	Silk contribution	Verification status; selected quantitative descriptor / NR
Release-mediated	Bakadia et al., 2023 [54]	PVA/sericin/azithromycin hydrogel	Sericin is a co-network with PVA	Azithromycin	Staphylococcus aureus, Pseudomonas aeruginosa, Escherichia coli, and Candida albicans.	Infected-animal model plus antibacterial evidence; coded C2 unless quantitative infected-tissue burden directly supports C4.	Deliberately infected mouse full-thickness burn model (10% total body surface area); tissue-CFU reduction was not used for C4 coding.	C2	S0: antimicrobial effect is additive-dominated; SF/SS mainly provides matrix, carrier, or co-network function.	FULL-TEXT VERIFIED. Infected mouse full-thickness burn model; 10% TBSA. Cross-study-comparable tissue CFU: NR in harmonized extraction.
Release-mediated	Li et al., 2025 [55]	Acetic-acid-induced sericin/ciprofloxacin hydrogel	Sericin forms the acid-induced network	Ciprofloxacin	Staphylococcus aureus and Pseudomonas aeruginosa	Infected-animal model plus antibacterial evidence; coded C2 unless quantitative infected-tissue burden directly supports C4.	Infected wound model; bacterial reduction was reported; quantitative tissue-burden details were not used for C4 coding.	C2	S0: antimicrobial effect is additive-dominated; SF/SS mainly provides matrix, carrier, or co-network function.	FULL-TEXT VERIFIED. NR in harmonized quantitative extraction; endpoint category and major limitation retained for cross-study comparison.
Release-mediated	Yin et al., 2022 [60]	SF/rhein hydrogel	SF is the drug-carrier matrix	Rhein	Burn-wound bacterial panel; strain identifiers NR.	Infected-animal model plus antibacterial evidence; coded C2 unless quantitative infected-tissue burden directly supports C4.	Bacteria-infected burn-wound model; wound repair was reported, while quantitative infected-tissue burden was not used for C4 coding.	C2	S0: antimicrobial effect is additive-dominated; SF/SS mainly provides matrix, carrier, or co-network function.	FULL-TEXT VERIFIED. Water content ~90%; water adsorption >2× own weight. Quantitative infected-tissue CFU: NR in harmonized extraction.
Release-mediated	Babaluei et al., 2023 [61]	SF/CMC/agarose/GO@PDA/vancomycin hydrogel	SF is a co-network in the injectable matrix	PDA-functionalized graphene oxide and vancomycin	MRSA	Retracted record; not used for evidence grading.	Excluded from animal/effect synthesis.	NG — retracted	NG — retracted	RETRACTED by the journal; excluded from all quantitative synthesis, claim-ceiling grading, and silk-contribution grading.

Mechanism	Reference	Composition	Structural SF/SS role	Direct antibacterial determinant	Microorganism(s)	Standardized microbial endpoint	Animal infection evidence	Claim ceiling	Silk contribution	Verification status; selected quantitative descriptor / NR
Release-mediated	Jafarbeglou et al., 2024 [62]	SF/chitosan-thiourea/PLGA(vancomycin+quercetin)	SF is a scaffold co-network	Vancomycin and quercetin	MRSA	Infected-animal model plus antibacterial evidence; coded C2 unless quantitative infected-tissue burden directly supports C4.	Chronic MRSA osteomyelitis model; efficacy was assessed with osteomyelitis outcome scores; quantitative bone/tissue CFU was not used for C4 coding.	C2	S0: antimicrobial effect is additive-dominated; SF/SS mainly provides matrix, carrier, or co-network function.	FULL-TEXT VERIFIED. PLGA nanoparticles ~206 nm; quercetin loading ~87%; sustained release >20 d. Quantitative bone/tissue CFU: NR in harmonized extraction.
Release-mediated	Phewchan et al., 2024 [63]	SF/calcium-phosphate/methylcellulose/vancomycin thermogel	SF is a thermogel co-component	Vancomycin	Gram-positive bacteria	Quantitative viable-bacteria reduction in vitro (C2).	Bone-infection-oriented local-delivery application; animal infection evidence was not used for claim coding.	C2	S0: antimicrobial effect is additive-dominated; SF/SS mainly provides matrix, carrier, or co-network function.	FULL-TEXT VERIFIED. NR in harmonized quantitative extraction; endpoint category and major limitation retained for cross-study comparison.
Release-mediated	Watchararot et al., 2025 [64]	SF/mupirocin hydrogel	SF is the drug-delivery matrix	Mupirocin	Staphylococcal skin pathogens; strain identifiers NR	Quantitative viable-bacteria reduction in vitro (C2).	Wound/irritation model; infection evidence was not used for claim coding.	C2	S0: antimicrobial effect is additive-dominated; SF/SS mainly provides matrix, carrier, or co-network function.	FULL-TEXT VERIFIED. NR in harmonized quantitative extraction; endpoint category and major limitation retained for cross-study comparison.
Release-mediated	Jing Y et al., 2023 [92]	SF/alginate/curcumin-nanoparticle hydrogel	SF is co-load-bearing with alginate	Curcumin nanoparticles.	Escherichia coli and Staphylococcus aureus.	Quantitative viable-bacteria reduction in vitro (C2).	Repeatedly inoculated E. coli/S. aureus wound model; quantitative in vivo tissue burden was not reported in the harmonized map.	C2	S0: antimicrobial effect is additive-dominated; SF/SS mainly provides matrix, carrier, or co-network function.	FULL-TEXT VERIFIED. NR in harmonized quantitative extraction; endpoint category and major limitation retained for cross-study comparison.

Mechanism	Reference	Composition	Structural SF/SS role	Direct antibacterial determinant	Microorganism(s)	Standardized microbial endpoint	Animal infection evidence	Claim ceiling	Silk contribution	Verification status; selected quantitative descriptor / NR
Contact-active/anti-adhesive	Xu Z et al., 2021 [67]	SF/chitosan hydrogel	SF is a co-load-bearing polymer	Chitosan-associated antibacterial activity, the sustained-release experiment is a separate carrier function rather than proof of SF antibacterial acti…	Escherichia coli and Staphylococcus aureus.	Growth inhibition / zone / OD or another non-CFU endpoint (C1).	No infected-animal model.	C1	S0: antimicrobial effect is additive-dominated; SF/SS mainly provides matrix, carrier, or co-network function.	FULL-TEXT VERIFIED. NR in harmonized quantitative extraction; endpoint category and major limitation retained for cross-study comparison.
Contact-active/anti-adhesive	Zhang et al., 2022 [68]	SF/tannic acid/F127DA/quaternized chitosan	SF is a structural co-network	Quaternized chitosan and tannic acid	Gram-positive and Gram-negative strains; strain identifiers NR	Quantitative viable-bacteria reduction in vitro (C2).	Wound-repair model without a deliberately established infection; no C4 claim.	C2	S0: antimicrobial effect is additive-dominated; SF/SS mainly provides matrix, carrier, or co-network function.	FULL-TEXT VERIFIED. NR in harmonized quantitative extraction; endpoint category and major limitation retained for cross-study comparison.
Contact-active/anti-adhesive	Khodaei et al., 2024 [69]	Particulate SF/Pluronic/curcumin hydrogel	SF forms the particulate hydrogel matrix	Curcumin, Pluronic also affected bacterial adhesion	MRSA	Quantitative planktonic and adherent MRSA CFU; anti-adhesion/surface-burden endpoint (C2), not mature-biofilm eradication.	No animal infection model.	C2	S1: measured/functional silk-assisted structural, release, stabilization, or interfacial role; direct silk-dependent killing not established.	FULL-TEXT VERIFIED. Pluronic-containing formulation reduced planktonic MRSA >99.981% and adherent MRSA >99.920%; curcumin-containing formulation reached up to ~99.999% reduction.
Contact-active/anti-adhesive	Chen et al., 2025 [70]	Antibiotic-free sericin/PATS composite hydrogel	Sericin is the principal/composite hydrogel component	Non-antibiotic active component (PATS), full chemical definition in article	Wound bacterial panel; strain identifiers NR	Inhibition-zone antibacterial endpoint (C1); non-infected wound closure is a separate host-repair endpoint.	Non-infected rat full-thickness skin-defect model; in vivo wound closure is separated from antibacterial evidence.	C1	S1: measured/functional silk-assisted structural, release, stabilization, or interfacial role; direct silk-dependent killing not established.	FULL-TEXT VERIFIED. Inhibition zones: E. coli 4.00±1.23 mm; S. aureus 6.88±1.04 mm. Non-infected wound closure ~85.85±3.65% at day 14.

Mechanism	Reference	Composition	Structural SF/SS role	Direct antibacterial determinant	Microorganism(s)	Standardized microbial endpoint	Animal infection evidence	Claim ceiling	Silk contribution	Verification status; selected quantitative descriptor / NR
Contact-active/anti-adhesive	Wu et al., 2026 [71]	SF/tannic-acid/polyphenol composite hydrogel	SF is the principal composite matrix	Tannic-acid/polyphenol-based antibacterial and antioxidant functionality; exact active determinant not isolated.	S. aureus/E. coli; strain identifiers NR	Quantitative viable-bacteria reduction in vitro (C2).	Wound-repair model; deliberately infected design was not used for claim coding.	C2	S1: measured/functional silk-assisted structural, release, stabilization, or interfacial role; direct silk-dependent killing not established.	FULL-TEXT VERIFIED. NR in harmonized quantitative extraction; endpoint category and major limitation retained for cross-study comparison.
Contact-active/anti-adhesive	El-Samad et al., 2022 [73]	Carboxymethyl cellulose/sericin hydrogel	Sericin is a co-network/bioactive component	No added classical antibiotic, claimed intrinsic CMC/sericin activity	Bacterial strains NR in harmonized extraction	Growth inhibition / zone / OD or another non-CFU endpoint (C1).	Diabetic wound model; deliberate infection was not used for claim coding.	C1	S0: antimicrobial effect is additive-dominated; SF/SS mainly provides matrix, carrier, or co-network function.	FULL-TEXT VERIFIED. NR in harmonized quantitative extraction; endpoint category and major limitation retained for cross-study comparison.
Metal-ion / catalytic	Tao et al., 2021 [47]	Sericin/alginate/Ag NP semi-IPN	Sericin is a functional semi-IPN component	In situ reduced Ag nanoparticles	Gram-positive and Gram-negative test strains; strain identifiers NR.	Quantitative viable-bacteria reduction in vitro (C2).	Wound-healing model; deliberate infection was not used for claim coding.	C2	S0: antimicrobial effect is additive-dominated; SF/SS mainly provides matrix, carrier, or co-network function.	FULL-TEXT VERIFIED. NR in harmonized quantitative extraction; endpoint category and major limitation retained for cross-study comparison.
Metal-ion / catalytic	Zhu et al., 2022 [49]	Methacrylated sericin/Ag cryogel	Sericin forms the cryogel network	In situ reduced/crosslinking silver species	Broad Gram-positive/Gram-negative panel	Quantitative viable-bacteria reduction in vitro (C2).	Wound/hemostasis model; deliberately infected design was not used for claim coding.	C2	S1: measured/functional silk-assisted structural, release, stabilization, or interfacial role; direct silk-dependent killing not established.	FULL-TEXT VERIFIED. NR in harmonized quantitative extraction; endpoint category and major limitation retained for cross-study comparison.
Metal-ion / catalytic	Laomeephol et al., 2025 [50]	Ag^+-crosslinked sericin hydrogel	Sericin forms the Ag^+-crosslinked network	Ag^+ serves as crosslinker and antimicrobial agent	E. coli, P. aeruginosa, S. aureus and broader panel	Quantitative viable-bacteria reduction in vitro (C2).	No infected-animal model.	C2	S1: measured/functional silk-assisted structural, release, stabilization, or interfacial role; direct silk-dependent killing not established.	FULL-TEXT VERIFIED. NR in harmonized quantitative extraction; endpoint category and major limitation retained for cross-study comparison.

Mechanism	Reference	Composition	Structural SF/SS role	Direct antibacterial determinant	Microorganism(s)	Standardized microbial endpoint	Animal infection evidence	Claim ceiling	Silk contribution	Verification status; selected quantitative descriptor / NR
Metal-ion / catalytic	Mao Y et al., 2023 [65]	SilMA/Ag@MSN-BMP-2 hydrogel	SilMA is the photo-crosslinked matrix	Ag^{+} embedded in mesoporous silica nanoparticles, BMP-2 is the osteogenic cargo.	Escherichia coli and Staphylococcus aureus.	Growth inhibition / zone / OD or another non-CFU endpoint (C1).	Non-inoculated incision and aseptic bone defect with postoperative penicillin; no infected-animal antibacterial claim.	C1	S0: antimicrobial effect is additive-dominated; SF/SS mainly provides matrix, carrier, or co-network function.	FULL-TEXT VERIFIED. NR in harmonized quantitative extraction; endpoint category and major limitation retained for cross-study comparison.
Metal-ion / catalytic	Zheng et al., 2022 [75]	SF/Ga^{3+} hydrogel	SF is the ion-induced matrix	Ga^{3+}	Pseudomonas aeruginosa	Quantitative viable-bacteria reduction in vitro (C2).	P. aeruginosa-infected rat wound (100 µL of 10^8 CFU/mL inoculum); in vivo assessment emphasized wound closure rather than quantitative tissue CFU.	C2	S1: measured/functional silk-assisted structural, release, stabilization, or interfacial role; direct silk-dependent killing not established.	FULL-TEXT VERIFIED. Rat wounds inoculated with 100 µL of 10^8 CFU/mL P. aeruginosa; quantitative tissue CFU: NR in harmonized extraction.
Metal-ion / catalytic	Zhu et al., 2022 [76]	MOF-bridged SF hydrogel	SF is the principal MOF-bridged network	Metal-organic framework with antibacterial cargo/function	Bacterial panel includes common wound pathogens; strain identifiers NR	Adherent-bacteria or biofilm-associated endpoint; coded C2 unless a pre-established viable-biofilm design was directly documented.	Wound-care animal model; animal infection status was not used for claim coding.	C2	S0: antimicrobial effect is additive-dominated; SF/SS mainly provides matrix, carrier, or co-network function.	FULL-TEXT VERIFIED. NR in harmonized quantitative extraction; endpoint category and major limitation retained for cross-study comparison.
Metal-ion / catalytic	Yang et al., 2022 [77]	SF/tannin/ZnO nanocomposite	SF is the structural matrix	ZnO nanoparticles and tannin	S. aureus/E. coli panel reported in article	Quantitative viable-bacteria reduction in vitro (C2).	No infected-animal model.	C2	S0: antimicrobial effect is additive-dominated; SF/SS mainly provides matrix, carrier, or co-network function.	FULL-TEXT VERIFIED. NR in harmonized quantitative extraction; endpoint category and major limitation retained for cross-study comparison.

Mechanism	Reference	Composition	Structural SF/SS role	Direct antibacterial determinant	Microorganism(s)	Standardized microbial endpoint	Animal infection evidence	Claim ceiling	Silk contribution	Verification status; selected quantitative descriptor / NR
Metal-ion / catalytic	Li et al., 2023 [78]	SF/H_2O_2-generating nanozyme hydrogel	SF forms the nanozyme hydrogel scaffold	Catalytic nanozyme system generating/supplying H_2O_2	Bacterial panel NR in harmonized extraction	Adherent-bacteria or biofilm-associated endpoint; coded C2 unless a pre-established viable-biofilm design was directly documented.	Animal infection status was not used for claim coding; antibacterial ceiling derives from the in vitro viable/biofilm-associated endpoint.	C2	S0: antimicrobial effect is additive-dominated; SF/SS mainly provides matrix, carrier, or co-network function.	FULL-TEXT VERIFIED. NR in harmonized quantitative extraction; endpoint category and major limitation retained for cross-study comparison.
Metal-ion / catalytic	Yang et al., 2024 [79]	Puerarin/SF/Ga^{3+} nanofibrillar hydrogel	SF forms the self-assembled nanofibrillar matrix	Gallium ions, puerarin	Pseudomonas and/or common wound pathogens	Infected-animal model plus antibacterial evidence; coded C2 unless quantitative infected-tissue burden directly supports C4.	Deliberately infected mouse-wound model; 100% wound closure by day 10 was reported; quantitative tissue-CFU reduction was not used for C4 coding.	C2	S1: measured/functional silk-assisted structural, release, stabilization, or interfacial role; direct silk-dependent killing not established.	FULL-TEXT VERIFIED. Ga^{3+} concentration 0.05% identified as an effective formulation; infected-wound closure reached 100% on day 10. Tissue CFU: NR in harmonized extraction.
Metal-ion / catalytic	Lalebeigi et al., 2024 [80]	Agar/tragacanth/SF/Zn-MOF hydrogel	SF is a reinforcing co-polymer	Zn-based MOF	Bacterial panel NR in harmonized extraction	Quantitative viable-bacteria reduction in vitro (C2).	No established animal infection model.	C2	S0: antimicrobial effect is additive-dominated; SF/SS mainly provides matrix, carrier, or co-network function.	FULL-TEXT VERIFIED. NR in harmonized quantitative extraction; endpoint category and major limitation retained for cross-study comparison.
Metal-ion / catalytic	Wiita et al., 2024 [81]	Silk microgels/selenium nanoparticles	Silk forms the antimicrobial microgel scaffold	Selenium nanoparticles	Bacterial and fungal panel	Quantitative viable-bacteria reduction in vitro (C2).	No infected-animal model.	C2	S0: antimicrobial effect is additive-dominated; SF/SS mainly provides matrix, carrier, or co-network function.	FULL-TEXT VERIFIED. NR in harmonized quantitative extraction; endpoint category and major limitation retained for cross-study comparison.
Metal-ion / catalytic	Li et al., 2025 [82]	Konjac glucomannan/SF/AgNP hydrogel	SF is a structural co-network	Silver nanoparticles	Wound bacteria	Quantitative viable-bacteria reduction in vitro (C2).	Wound model; quantitative infected-tissue burden was not used for claim coding.	C2	S0: antimicrobial effect is additive-dominated; SF/SS mainly provides matrix, carrier, or co-network function.	FULL-TEXT VERIFIED. NR in harmonized quantitative extraction; endpoint category and major limitation retained for cross-study comparison.

Mechanism	Reference	Composition	Structural SF/SS role	Direct antibacterial determinant	Microorganism(s)	Standardized microbial endpoint	Animal infection evidence	Claim ceiling	Silk contribution	Verification status; selected quantitative descriptor / NR
Metal-ion / catalytic	Eivazzadeh-Keihan et al., 2020 [83]	Alginate/PVA/SF/Mg(OH)$_2$-nanorod scaffold	SF is a reinforcing co-polymer	Mg(OH)$_2$ nanorods	Bacterial species not specified in the accessible abstract	Abstract/database-level description only; not used for detailed evidence grading.	No infected-animal model.	NG — abstract/database only	NG — abstract/database only	ABSTRACT/DATABASE ONLY; retained for audit transparency and descriptive indexing, but excluded from detailed C/S grading and graded-study percentages.
Light-responsive	Yan et al., 2024 [58]	SF/Ag@reduced-graphene-oxide hydrogel	SF is the composite matrix	Ag and rGO photothermal activity	S. aureus/E. coli and wound pathogens	Infected-animal model plus antibacterial evidence; coded C2 unless quantitative infected-tissue burden directly supports C4.	Bacterial-stained/full-thickness infected-wound model; in vivo repair/antibacterial benefit reported; quantitative tissue-CFU reduction was not used for C4 coding.	C2	S1: measured/functional silk-assisted structural, release, stabilization, or interfacial role; direct silk-dependent killing not established.	FULL-TEXT VERIFIED. NR in harmonized quantitative extraction; endpoint category and major limitation retained for cross-study comparison.
Light-responsive	Yao et al., 2025 [84]	SF/Au@MOF phototherapy hydrogel	SF is the hydrogel matrix	Au@MOF photothermal/photodynamic platform	Wound bacteria	Infected-animal model plus antibacterial evidence; coded C2 unless quantitative infected-tissue burden directly supports C4.	Infected-wound phototherapy model; quantitative tissue-burden data were not used for C4 coding.	C2	S1: measured/functional silk-assisted structural, release, stabilization, or interfacial role; direct silk-dependent killing not established.	FULL-TEXT VERIFIED. NR in harmonized quantitative extraction; endpoint category and major limitation retained for cross-study comparison.
Light-responsive	Cheng et al., 2024 [87]	SF-coated liquid-metal hydrogel	SF stabilizes/coats the liquid-metal phase	Liquid-metal photothermal agent	Wound bacteria; strain identifiers NR	Infected-animal model plus antibacterial evidence; coded C2 unless quantitative infected-tissue burden directly supports C4.	Infected-wound model with NIR photothermal treatment; quantitative tissue-CFU reduction was not used for C4 coding.	C2	S1: measured/functional silk-assisted structural, release, stabilization, or interfacial role; direct silk-dependent killing not established.	FULL-TEXT VERIFIED. NR in harmonized quantitative extraction; endpoint category and major limitation retained for cross-study comparison.

Mechanism	Reference	Composition	Structural SF/SS role	Direct antibacterial determinant	Microorganism(s)	Standardized microbial endpoint	Animal infection evidence	Claim ceiling	Silk contribution	Verification status; selected quantitative descriptor / NR
Light-responsive	Yao et al., 2026 [88]	Dual-network SF/W18O49-x@Au hydrogel	SF forms the dual-network matrix	W18O49-x@Au photothermal/photodynamic agent	Wound bacteria	Infected-animal model plus antibacterial evidence; coded C2 unless quantitative infected-tissue burden directly supports C4.	Infected rat-wound phototherapy model; quantitative tissue-burden data were not used for C4 coding.	C2	S1: measured/functional silk-assisted structural, release, stabilization, or interfacial role; direct silk-dependent killing not established.	FULL-TEXT VERIFIED. NR in harmonized quantitative extraction; endpoint category and major limitation retained for cross-study comparison.
Light-responsive	Yan et al., 2025 [89]	SF/Ag@PDA/curcumin-PDA hydrogel	SF is the composite matrix	Ag@PDA and Cur-PDA nanoparticles	Staphylococcus aureus and Escherichia coli	Infected-animal model plus antibacterial evidence; coded C2 unless quantitative infected-tissue burden directly supports C4.	Infected-wound model with NIR-triggered treatment; quantitative tissue-CFU reduction was not used for C4 coding.	C2	S1: measured/functional silk-assisted structural, release, stabilization, or interfacial role; direct silk-dependent killing not established.	FULL-TEXT VERIFIED. NR in harmonized quantitative extraction; endpoint category and major limitation retained for cross-study comparison.
Multimodal / composite	Ke et al., 2020 [26]	Self-healing natural-polymer/SF bioadhesive	SF-containing dynamic adhesive network	Antibacterial activity arises from composite chemistry, exact active moiety NR in harmonized extraction	Staphylococcus aureus and Escherichia coli.	Quantitative viable-bacteria reduction in vitro (C2).	Wound-repair model; deliberate infection was not used for claim coding.	C2	S1: measured/functional silk-assisted structural, release, stabilization, or interfacial role; direct silk-dependent killing not established.	FULL-TEXT VERIFIED. NR in harmonized quantitative extraction; endpoint category and major limitation retained for cross-study comparison.
Multimodal / composite	Guo et al., 2023 [56]	SFMA/ε-polylysine/MnO_2 hydrogel	SFMA is the photo-crosslinked matrix	ε-Polylysine (EPL) and MnO_2 nanoparticles	Staphylococcus aureus; additional strains may be tested	Infected-animal model plus antibacterial evidence; coded C2 unless quantitative infected-tissue burden directly supports C4.	S. aureus-infected diabetic-wound model; antibacterial benefit reported; quantitative tissue-CFU reduction was not used for C4 coding.	C2	S1: measured/functional silk-assisted structural, release, stabilization, or interfacial role; direct silk-dependent killing not established.	FULL-TEXT VERIFIED. NR in harmonized quantitative extraction; endpoint category and major limitation retained for cross-study comparison.

Mechanism	Reference	Composition	Structural SF/SS role	Direct antibacterial determinant	Microorganism(s)	Standardized microbial endpoint	Animal infection evidence	Claim ceiling	Silk contribution	Verification status; selected quantitative descriptor / NR
Multimodal / composite	Zeng et al., 2023 [57]	Thiolated gelatin/SilMA/NO-generating nanoparticles	SilMA is a co-network with thiolated gelatin	Metal–polyphenol nanoparticles catalyzing nitric oxide release	MRSA	Infected-animal model plus antibacterial evidence; coded C2 unless quantitative infected-tissue burden directly supports C4.	MRSA-infected wound model; antibacterial benefit reported; quantitative tissue-CFU reduction was not used for C4 coding.	C2	S1: measured/functional silk-assisted structural, release, stabilization, or interfacial role; direct silk-dependent killing not established.	FULL-TEXT VERIFIED. NR in harmonized quantitative extraction; endpoint category and major limitation retained for cross-study comparison.
Multimodal / composite	Sun M et al., 2024 [59]	SilkMA/CaO_2/catalase/AgNP microneedle patch	SilkMA is the load-bearing microneedle matrix	Ag nanoparticles, CaO_2/catalase is primarily the oxygen-generating module.	Escherichia coli and Staphylococcus aureus.	Growth inhibition / zone / OD or another non-CFU endpoint (C1).	Diabetic wound without deliberate inoculation; surface-swab culture only, not a quantitative infected-tissue burden.	C1	S1: measured/functional silk-assisted structural, release, stabilization, or interfacial role; direct silk-dependent killing not established.	FULL-TEXT VERIFIED. NR in harmonized quantitative extraction; endpoint category and major limitation retained for cross-study comparison.
Multimodal / composite	Schnaider et al., 2020 [74]	SF nanofibrillar microgels/Ag nanoparticles	SF forms the microgel scaffold	Ag nanoparticles	Bacterial species not fully listed in abstract	Abstract/database-level description only; not used for detailed evidence grading.	Murine surgical-site infection model; animal efficacy reported; quantitative tissue-burden data were not used for C4 coding.	NG — abstract/database only	NG — abstract/database only	ABSTRACT/DATABASE ONLY; retained for audit transparency and descriptive indexing, but excluded from detailed C/S grading and graded-study percentages.
Multimodal / composite	Zhang et al., 2021 [85]	Sericin/chitosan/Ag@MOF/graphene oxide	Sericin is a co-network/bioactive component	Ag@MOF and chitosan	S. aureus and E. coli or comparable Gram+/Gram− panel	Quantitative viable-bacteria reduction in vitro (C2).	Wound-repair model; deliberate infection was not used for claim coding.	C2	S0: antimicrobial effect is additive-dominated; SF/SS mainly provides matrix, carrier, or co-network function.	FULL-TEXT VERIFIED. NR in harmonized quantitative extraction; endpoint category and major limitation retained for cross-study comparison.
Multimodal / composite	Yan et al., 2023 [86]	SF hydrogel with sericin-decorated curcumin/Ag nanoparticles	SF is the matrix; sericin modifies nanoparticles	Silver nanoparticles and curcumin	Common wound bacteria; strain identifiers NR.	Quantitative viable-bacteria reduction in vitro (C2).	S. aureus-infected mouse wound model; quantitative infected-tissue burden was not used for C4 coding.	C2	S0: antimicrobial effect is additive-dominated; SF/SS mainly provides matrix, carrier, or co-network function.	FULL-TEXT VERIFIED. NR in harmonized quantitative extraction; endpoint category and major limitation retained for cross-study comparison.

Mechanism	Reference	Composition	Structural SF/SS role	Direct antibacterial determinant	Microorganism(s)	Standardized microbial endpoint	Animal infection evidence	Claim ceiling	Silk contribution	Verification status; selected quantitative descriptor / NR
Multimodal / composite	Chu et al., 2023 [90]	Chitosan/sericin/Ag^{+}/lupeol hydrogel	Sericin is a functional co-component	Ag^{+} and lupeol, chitosan contributes	Gram-positive and Gram-negative bacteria	Infected-animal model plus antibacterial evidence; coded C2 unless quantitative infected-tissue burden directly supports C4.	Wound model with bacterial-proliferation assessment; quantitative infected-tissue burden was not used for C4 coding.	C2	S0: antimicrobial effect is additive-dominated; SF/SS mainly provides matrix, carrier, or co-network function.	FULL-TEXT VERIFIED. NR in harmonized quantitative extraction; endpoint category and major limitation retained for cross-study comparison.
Multimodal / composite	Wang et al., 2025 [91]	Visualizable antimicrobial sericin hydrogel	Sericin is the principal hydrogel network	Antimicrobial functional groups/payload; exact active chemistry NR in harmonized extraction.	Wound bacteria	Infected-animal model plus antibacterial evidence; coded C2 unless quantitative infected-tissue burden directly supports C4.	Diabetic-wound model; deliberate infection was not used for claim coding.	C2	S1: measured/functional silk-assisted structural, release, stabilization, or interfacial role; direct silk-dependent killing not established.	FULL-TEXT VERIFIED. NR in harmonized quantitative extraction; endpoint category and major limitation retained for cross-study comparison.
Multimodal / composite	Han et al., 2025 [93]	SilMA/silver–gallic-acid nanoparticle hydrogel	SilMA is the photocrosslinked matrix	Silver–gallic acid nanoparticles	Wound bacteria	Quantitative viable-bacteria reduction in vitro (C2).	Diabetic wound model; deliberate infection was not used for claim coding.	C2	S0: antimicrobial effect is additive-dominated; SF/SS mainly provides matrix, carrier, or co-network function.	FULL-TEXT VERIFIED. NR in harmonized quantitative extraction; endpoint category and major limitation retained for cross-study comparison.

Notes: Verification status is reported in the final column. Detailed claim ceilings (C0–C5) and silk-contribution categories (S0–S3) are assigned only to full-text-verified, non-retracted records. NG = not graded because full-text verification was insufficient or the record was excluded; NR = not available as a harmonizable numeric value in the present extraction. C4 requires a deliberately infected tissue model plus quantitative bacterial-burden measurement; wound closure, surface swab, histology, or an infected-model label alone does not establish C4. Antimicrobial and host-repair endpoints are interpreted independently.
